# Characterising the Metabolomic Diversity and Biological Potentials of Extracts from Different Parts of Two *Cistus* Species Using UHPLC-MS/MS and In Vitro Techniques

**DOI:** 10.3390/pathogens13090795

**Published:** 2024-09-13

**Authors:** Shakeel Ahmed, Gokhan Zengin, Selami Selvi, Gunes Ak, Zoltán Cziáky, József Jekő, Maria J. Rodrigues, Luisa Custodio, Roberto Venanzoni, Giancarlo Angeles Flores, Gaia Cusumano, Paola Angelini

**Affiliations:** 1 Physiology and Biochemistry Laboratory, Department of Biology, Science Faculty, Selcuk University, Konya 42130, Turkey; shakeel.phytomedicine@gmail.com (S.A.); akguneselcuk@gmail.com (G.A.); 2 Department of Plant and Animal Production, Altınoluk Vocational School, Balıkesir University, Balıkesir 10870, Turkey; sselvi@balikesir.edu.tr; 3 Agricultural and Molecular Research and Service Institute, University of Nyíregyháza, 4400 Nyíregyháza, Hungary; cziaky.zoltan@nye.hu (Z.C.); jjozsi@gmail.com (J.J.); 4 Centre of Marine Sciences, University of Algarve, Campus of Gambelas, 8005-139 Faro, Portugal; mjrodrigues@ualg.pt (M.J.R.); lcustodio@ualg.pt (L.C.); 5 Department of Chemistry, Biology and Biotechnology, University of Perugia, 06122 Perugia, Italy; roberto.venanzoni@unipg.it (R.V.); giancarlo.angelesflores@unich.it (G.A.F.); gaia.cusumano@studenti.unipg.it (G.C.)

**Keywords:** *C. monspeliasis*, *C. parviflorus*, chemical profiling, antioxidants, enzyme inhibition

## Abstract

This study investigates the biochemical composition and biological properties of different parts (leaves, roots, and twigs) of two *Cistus* species (*Cistus monspeliasis* and *Cistus parviflorus*). The extracts were analysed using UHPLC-MS/MS to determine their chemical profiling. A range of antioxidant assays were performed to evaluate the extract’s antioxidant capabilities. The enzyme inhibition studies focused on acetylcholinesterase (AChE), butyrylcholinesterase (BChE), α-amylase, and α-glucosidase and tyrosinase. In addition, the study examined the antimicrobial effects on different bacteria and yeasts and evaluated the toxicity using the MTT assay. Quinic acid, citric acid, gallic acid, catechin, quercetin derivatives, kaempferol, myricetin, ellagic acid, prodelphinidins, procyanidins, scopoletin, and flavogallonic acid dilactone are the main bioactive compounds found in both species. In enzyme inhibition assays, *C. monspeliasis* roots exhibited significant activity against acetylcholinesterase (AChE) and butyrylcholinesterase (BChE), with the values of 2.58 ± 0.02 mg GALAE/g and 11.37 ± 1.93 mg GALAE/g, respectively. Cytotoxicity studies showed mostly weak toxicity, with some samples moderately reducing viability in RAW and HepG2 cells. These findings underscore the diverse biochemical profiles and bioactive potential of *Cistus* species, suggesting their utility as natural sources of antioxidants and enzyme inhibitors for pharmaceutical and nutraceutical development.

## 1. Introduction

In the past decade, there has been a substantial increase in the focus on bioactive substances derived from natural sources, specifically antioxidants, such as polyphenols [1,2]. Plants that possess antioxidants are utilised in semi-synthetic procedures as essential primary substances to prepare new formulations. Extracts obtained by the plants are also conventionally employed in folkloric medicine to alleviate diverse ailments and diseases owing to their phenolic composition [3]. Polyphenols are a broad category of natural compounds that have biological effects such as fighting against microbial infections, safeguarding the heart, inhibiting cancer growth, slowing ageing, protecting against oxidative damage, and reducing inflammation [4].

*Cistus* L. species, belonging to the Cistaceae family, are enduring perennial and dicotyledonous shrubs. They are distributed in the Mediterranean climate. Of the 21 species, 16 were found across a large geographical area of Turkey [5,6]. In the Mediterranean region, traditional medicine has utilised infusions and extracts derived from *Cistus* species to address a range of health conditions including rheumatic pain, skin inflammation, wound healing, the common cold, and digestive disorders [6,7,8].

The Mediterranean area is home to 21 species of the genus *Cistus* L., while *C. monspeliensis* L., *C. laurifolius* L., *C. creticus* L., *C. salviifolius* L., and *C. parviflorus* Lam. belong to Turkey origin [9,10]. The crude extracts and essential oils (EOs) derived from cistus species have demonstrated efficacy in treating skin problems, diabetes, wound healing, snakebites, oedema, high fever, diarrhoea, peptic ulcers, inflammation, rheumatism, and urinary infections in Anatolian folk medicine [6,11,12]. *Cistus* essential oil (EO) has received approval from the Food and Drug Administration (FDA) as a food additive and flavouring agent. Given the growing fascination with natural products and the advantageous properties of *Cistus* species, essential oil, labdanum, bee pollen, and tea are making a noteworthy impact in the realm of herbal products. According to the findings, *C. monspeliensis* can enhance energy metabolic pathways in human intestinal epithelial cells [13].

Recently, there has been an increasing interest in the biological effects associated with the traditional uses of these plants, specifically their ability to combat microbes, prevent oxidation, inhibit tumour growth, fight viruses, reduce inflammation, and prevent ulcers. The abundant chemical composition of these plants has significantly contributed to a growing number of research projects. Originally, research was mostly centred around the resin excreted by the glandular trichomes of leaves, primarily because of its application in perfumery [14]. Subsequently, numerous studies have conducted a comprehensive analysis of the phytochemicals found in different extracts of distinct *Cistus* species [15,16,17,18].

After thoroughly examining multiple scientific articles on different *Cistus* species and comparing them with our research, a consistent range of chemicals has been established in various investigations [19,20,21]. The rich profile of bioactive components found in *Cistus* species includes flavonoids, particularly derivatives of apigenin, quercetin, and kaempferol, as well as tannins like ellagic acid, sterols, phenolic acids, and lignan glycosides. The profile of these kinds of chemicals from different species of *Cistus* suggests potential therapeutic applications [9,22]. According to previous research, chemical examination of various tissues from different species of *Cistus* revealed distinct chemical classes, such as diterpenes, which are typically found in *C. monspeliensis* L. and *C. libanotis* L. [23]. These plants are known to contain many chemicals from different chemical classes, including flavonoids, coumarins, terpene derivatives, and hydrocarbons [24,25]. Multiple studies have documented the presence of phytochemicals in extracts obtained from various *Cistus* species across different geographical areas [26]. Therefore, it can be broadly asserted that our *Cistus* species possess a multitude of bioactive chemicals that have been extensively reported in the scientific literature on other *Cistus* species.

The objective of this study was to examine the chemical makeup of methanolic extracts derived from different parts of *C. monspeliensis*, and *C. parviflorus*, and assess their antioxidant and different inhibitory effects against multiple enzymes important to human health. The extracts underwent UHPLC-MS/MS analysis, and their antioxidant capacity was evaluated using several in vitro assays that elucidate distinct modes of action. More precisely, the assessment of radical scavenging activities was conducted by the utilisation of ABTS and DPPH assays. Additionally, CUPRAC and metal chelating assays were performed to obtain a more detailed antioxidant potential of the extracts. In addition, the FRAP assay measured the extracts’ effect on iron, a crucial ion in oxidation reactions. Furthermore, the study sought to determine the efficacy of the extracts against the inhibitory activities of acetylcholinesterase (AChE) and butyrylcholinesterase (BChE), α-amylase and α-glucosidase, and a potential candidate for tyrosinase inhibitor. Additionally, the antimicrobial and cytotoxic potential of all the extracts were evaluated against bacterial species, yeast species, human hepatocarcinoma (HepG2), murine macrophages (RAW 264.7), and mouse bone marrow stromal (S17) cell lines.

## 2. Materials and Methods

### 2.1. Plant Collection

In 2021, plant materials were gathered from the West Region of Anatolia, Turkey. Detailed information is provided below. Dr. Selami Selvi performed the taxonomic identification, and a voucher specimen was stored in the herbarium of Balıkesir University. Leaves, twigs, and roots were carefully separated, dried in the shade at room temperature, ground, and stored in darkness.

1. *C. parviflorus* Lam. Turkey; C2 Mugla: Gokova Gulf, Akbuk Bay, macchie, 37°1′40.13″ N, 28°5′33.10″ E, 50 m, Voucher No.: SV 3439.

2. *C. monspeliensis* L. Turkey; B1 İzmir; Çeşme, macchie, 38°21′55.09″ N, 26°52′37.59″ E, 33 m, Voucher No.: SV 2955.

### 2.2. Plant Extract Preparation

Methanol was used to prepare the extracts. Approximately 10 g of the sample was soaked in 200 mL of methanol for 24 h at room temperature. The methanol was subsequently evaporated under reduced pressure, and the extracts were kept at 4 °C until further analysis.

### 2.3. Assay for Total Phenolic and Flavonoid Contents

Following the procedures specified by [27], total phenolics and flavonoids were measured. Gallic acid (GA) and rutin (RE) were employed as references in the experiments, with the results presented as gallic acid equivalents (GAEs) and rutin equivalents.

### 2.4. UHPLC-MS/MS Analysis

Analysis of different extracts was carried out on liquid chromatography coupled with mass spectrometry (UHPLC-MS/MS) using a system in which a UHPLC (Dionex Ultimate 3000RS) system was equipped with a Mass Spectrometer (Q-Exactive Orbitrap, Thermo, Waltham, MA, USA).

Before the analysis, extracts were filtered through 0.22 μm PTFE filter membrane (Labex Ltd., Budapest, Hungary). In order to achieve chromatographic separation, 2 µL of each sample was injected into the HPLC system equipped with a reverse-phase C-18 column (Accucore C18 (100 mm × 2.1, mm i. d., 2.6 μm, Thermo). The column was thermostated at 25 °C (±1 °C). The elution was carried out at a flow rate of 0.2 mL/min using gradient elution. The solvents used were water (A) and methanol (B). Both were acidified with 0.1% formic acid. Elution was performed using the following gradients: isocratic 5% B (0–3 min), a linear gradient increasing from 5% B to 100% (3–43 min), 100% B (43–61 min), a linear gradient decreasing from 100% B to 5% (61–62 min), and 5% B (62–70 min). The total run time of analysis was 70 min.

The Thermo Q-Exactive Orbitrap mass spectrometer equipped with electrospray ionisation source was in positive or negative polarity at the resolving power of 70,000 (full MS, range: *m*/*z* 100–1500) and 35,000 (ddMS^2^). The ESI source parameters include ion spray voltage 4.0 kV in positive and 3.8 kV in negative mode; capillary temperature 320 °C; S-lens RF level 50 V; auxiliary gas: N2 (purity > 95%), heater temperature 300 °C. The data were acquired in full MS-ddMS^2^ mode by using Xcalibur 3.1 software. The full MS-ddMS^2^ mode provided a full MS with MS/MS spectrum simultaneously in a single LC run. The full MS spectrum provided information about the intact molecular ion (e.g., M^+^, [M + H]^+^ , [M − H]^−^), while the ddMS^2^ discovery generates the product ion spectra. The acquired data were processed by using TraceFinder 3.1 software (Thermo Fisher Scientific). Two runs were made with each sample, spectra were recorded separately in positive and negative mode. This method helped the identification of compounds in comparison with databases, and peak identification was also based on the comparison of the chromatographic data with standards, the exact molecular mass/adducts, fragmentation patterns, isotopic distributions and comparison with our own tandem mass spectral library (MS/MS). As can be seen from the data in the Tables (next chapter), a large number of components, structural isomers were tentatively identified. All samples were measured in both positive and negative ionisation mode; the data recorded in the negative mode were more suitable for identifying the components.

### 2.5. Assays for In Vitro Antioxidant Capacity

Following the methods described by [28], antioxidant tests were performed. The findings of the DPPH, ABTS radical scavenging, CUPRAC, and FRAP tests were quantified in milligrams of Trolox equivalents (TEs) per gram of extract. The antioxidant potential, as indicated by the phosphomolybdenum (PBD) assay, was quantified in millimoles of Trolox equivalents (TEs) per gram of extract. The metal chelating activity (MCA) was expressed as milligrams of disodium edetate equivalents (EDTAEs) per gram of extract.

### 2.6. Inhibitory Effects against Some Key Enzymes

According to established protocols [28], enzyme inhibition experiments were conducted on the samples. Amylase and glucosidase inhibition were quantified in acarbose equivalents (ACAEs) per gram of extract, while acetylcholinesterase (AChE) and butyrylcholinesterase (BChE) inhibition were indicated in milligrams of galanthamine equivalents (GALAEs) per gram of extract. Tyrosinase inhibition was assessed in milligrams of kojic acid equivalents (KAEs) per gram of extract.

### 2.7. Antimicrobial Activity

In vitro tests were conducted to evaluate the antimicrobial activity of *Cistus* extracts against a panel of four bacterial strains, both Gram-negative and Gram-positive: *Escherichia coli* (ATCC 10536), *Pseudomonas aeruginosa* (ATCC 15442), *Bacillus subtilis* (PeruMyc 6), and *Salmonella typhy* (PeruMyc 7). Additionally, these extracts were tested for antifungal properties against several yeast and dermatophyte species, including *Candida tropicalis* (YEPGA 6184), *C. albicans* (YEPGA 6379), *C. parapsilopsis* (YEPGA 6551), *Trichophyton mentagrophytes* (CCF 4823), *Trichophyton tonsurans* (CCF 4834), *Arthroderma quadrifidum* (CCF 5792), *Trichophyton mentagrophytes* (CCF 5930), *Arthroderma insingulare* (CCF 5417), and *Auxarthron ostraviense* (DB7).

*Candida parapsilosis* (ATCC 22019) and *C. krusei* (ATCC 6258) were used as quality control strains in antifungal tests, adhering to the protocols in CLSI documents M27-A3, M38-A2, M27-S4, and supplement M61. The PeruMycA culture collection at the DCBB, University of Perugia, Italy, maintains these voucher cultures and provides them upon request. The MIC of *Cistus* extracts was assessed within the range of 1.562–200 μg mL^−1^. Controls included Ciprofloxacin (Sigma) at 1.56–200 μg mL^−1^, Fluconazole (Sigma) at 0.063–16 μg mL^−1^, and Griseofulvin (Sigma) at 0.03–8 μg mL^−1^ [29].

The MIC endpoints for *Cistus* extracts were determined by the lowest concentration showing no visible growth. For Ciprofloxacin, Fluconazole, and Griseofulvin, these endpoints were the lowest concentrations that inhibited 80% of growth relative to the control [30,31].

### 2.8. Antibacterial/Antifungal Susceptibility Testing

Antibacterial susceptibility testing was conducted to determine the minimal inhibitory concentration (MIC) of *Cistus* extracts using a microdilution method, in accordance with the Clinical and Laboratory Standards Institute (CLSI) M07-A9 protocol [32]. Antifungal susceptibility testing for yeasts and filamentous fungi was conducted according to the guidelines specified in CLSI M27-A3 and M38-A2 protocols [31,32,33,34]. Detailed descriptions of these protocols are provided in the Supplementary Materials.

### 2.9. Cell Culture

The HepG2, RAW 264.7, and S17 cell lines, representing human hepatocarcinoma, murine macrophages, and mouse bone marrow stromal cells, respectively, were cultured in DMEM containing 10% foetal bovine serum, 2 mM of L-glutamine (1%), and penicillin (50 U/mL)/streptomycin (50 μg/mL) (1%), kept at 37 °C with 5% CO_2_ in a humidified atmosphere.

### 2.10. Determination of Cellular Viability

Cells were seeded in 96-well plates at a density of 5 × 10^3^ cells/well for HepG2 and S17, and 1 × 10^4^ cells/well for RAW 264.7. After incubating overnight, cells were treated with extracts at a concentration of 100 μg/mL for 72 h. Cells treated with 0.5% DMSO served as the control. Cellular viability was assessed using the MTT (3-(4,5-dimethylthiazol-2-yl)-2,5-diphenyltetrazolium bromide) assay, as previously described in our earlier paper [1]. The percentage of cellular viability was calculated relative to the DMSO (0.5%) control.

### 2.11. Statistical Analysis

The results were given as mean ± SD of three parallel experiments. Differences in extract levels among the extracts were assessed using ANOVA with Tukey’s test. The Pearson correlation test was employed to investigate the relationship between the total bioactive compounds in the tested extracts and their biological activities (antioxidant and enzyme inhibitory). GraphPad 9.0 was used for all analyses.

## 3. Results

### 3.1. Chemical Composition

A list of all compounds identified by UHPLC-MS/MS in all extracts of both *Citrus* species is present in Table 1 and Table 2. The details of the results and chromatograms are given in Supplementary Materials.

Both *C. monspeliensis* and *C. parviflorus* exhibit a notable range of chemical diversity in their leaves, twigs, and roots, containing various organic acids, phenolic acids, and flavonoids. Their leaves possess notable antioxidant qualities due to the presence of shared components such as quinic acid, citric acid, gallic acid, and gentisic acid. Flavonoids including catechin, epicatechin, quercetin derivatives, kaempferol, and myricetin improve their ability to prevent oxidation. Both species found in the twigs contain procyanidins and flavogallonic acid dilactone, which are renowned for their ability to block enzymes. Nevertheless, *C. parviflorus* stands out due to its distinct compounds such as esculetin and pheophytin A found in its leaves, as well as specific prodelphinidins and epigallocatechin present in its twigs. On the other hand, *C. monspeliensis* contains malic acid in its leaves and various oligomeric proanthocyanidins in its twigs. The roots of both plants contain a high concentration of phenolic acids. In addition, *C. parviflorus* also contains substances such as dimethoxy-trihydroxyflavone-O-hexoside and 3-O-methylellagic acid-4′-O-rhamnoside, which contribute to its unique medicinal properties [35].

The leaves of *C. parviflorus* contain a wide range of chemicals that belong to several chemical classes. Quinic acid and citric acid, which are well-known organic acids, are present and are highly regarded for their strong antioxidant effects [36]. The antioxidative activities of the leaves are enhanced by phenolic acids, including gallic acid and protocatechuic acid. The plant’s capacity to counteract oxidative stress is greatly enhanced by the presence of several flavonoids, such as catechin, epicatechin, quercetin derivatives, kaempferol, and myricetin. Esculetin and scopoletin, which are types of coumarins, provide supplementary therapeutic advantages, namely in terms of their anti-inflammatory properties. The leaves also contain ellagic acid and pheophytin A, which enhance the pharmacological profile.

*C. parviflorus* twigs also contain a wide variety of bioactive chemicals. Prodelphinidins and procyanidins, which are kinds of condensed tannins, are well known for their antioxidant and enzyme inhibitory effects [37,38]. These compounds are important components. Flavogallonic acid dilactone is a chemical that is well known for its bioactivity. Coumarins, such as scopoletin and its derivatives, including scopolin, can block enzymes, particularly tyrosinase [39]. The twigs’ extensive therapeutic effects are further enhanced by compounds such as catechol, methyl gallate, epigallocatechin, caffeic acid, and naringenin-6,8-di-C-glucoside [40].

The roots of *C. parviflorus* also contain a significant amount of useful chemicals. Phenolic acids, such as gentisic acid, gallic acid, and p-coumaric acid, are well known for their anti-inflammatory and antioxidant characteristics [41]. The roots also contain substantial quantities of procyanidin B isomers, which possess potential anti-diabetic properties [42]. Flavonoid glycosides, such as myricetin-O-glucoside and hyperoside, improve the therapeutic capacity [43]. The inclusion of scopoletin derivatives enhances the therapeutic efficacy of the roots. Additional notable substances found in the roots are dimethoxy-trihydroxyflavone-O-hexoside, astragalin, and 3-O-methylellagic acid-4′-O-rhamnoside, which contribute to the intricate chemical composition of the roots. The significant therapeutic potential of *C. parviflorus* is highlighted by the varied chemical composition found in its leaves, twigs, and roots.

Similarly, the leaves of *C. monspeliensis* display a wide range of chemicals belonging to different chemical groups. The compounds mentioned are quinic acid, malic acid, citric acid, gallic acid, and gentisic acid, all known for their ability to act as antioxidants [44]. The antioxidant properties of the leaves are greatly enhanced by the presence of flavonoids such as catechin, epicatechin, quercetin derivatives, kaempferol, and myricetin [45]. In addition, phenolic acids such as ellagic acid enhance the leaves’ ability to defend against oxidative stress.

*C. monspeliensis* contains a variety of bioactive substances in its twigs, such as procyanidins, a form of condensed tannin, and flavogallonic acid dilactone, which are renowned for their ability to inhibit enzymes. Scopoletin and its glucoside derivatives, which are coumarins, can potentially block tyrosinase. Additional noteworthy substances found in the twigs are catechol, methyl gallate, and several oligomeric proanthocyanidins. These compounds play a substantial role in the total bioactivity of the plant.

The roots of *C. monspeliensis* contain an abundance of bioactive chemicals. Phenolic acids, including gentisic acid, gallic acid, and p-coumaric acid, are recognised for their anti-inflammatory and antioxidant characteristics [41,46,47]. Flavonoid glycosides, such as myricetin-O-glucoside and hyperoside, offer supplementary advantages for one’s health. The roots also contain substantial quantities of procyanidins B isomers, which are condensed tannins with possible anti-diabetic properties. The roots also contain scopoletin derivatives, which enhance their medicinal potential, specifically in regulating glucose metabolism and insulin sensitivity [48,49]. Additional notable substances found in the roots are dimethoxy-trihydroxyflavone-O-hexoside, astragalin, and 3-O-methylellagic acid-4′-O-rhamnoside, which contribute to the intricate chemical composition of the roots. The wide range of chemical compounds found in the leaves, twigs, and roots of *C. monspeliensis* demonstrates significant potential for medicinal use.

**Figure 1:**
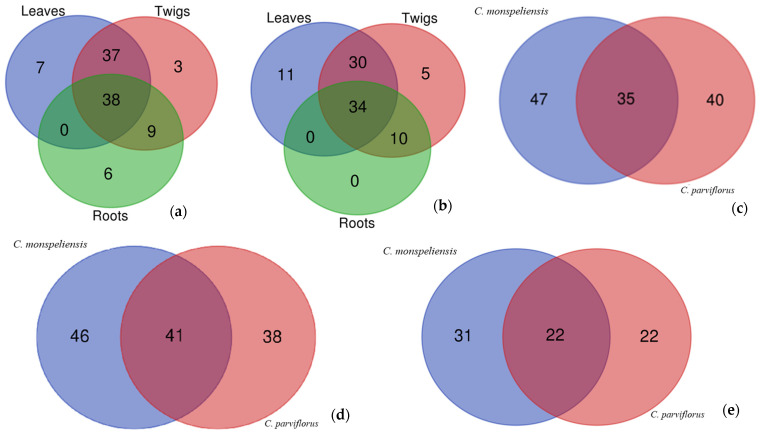
Venn diagrams based on the numbers of identified compounds in the tested extracts. (**a**): The parts of *Cistus monspeliasis*; (**b**): The parts of *Cistus parviflorus*; (**c**): Leaves extracts of both *Cistus* species; (**d**): twigs extracts of both *Cistus* species; (**e**): root extracts of both *Cistuss* species.

To gain more insight into the differences between the tested extracts in terms of the number of compounds identified, we created Venn diagrams. The results are shown in Figure 1. Figure 1a,b show the differences in different parts of the same *Cistus* species. Although most compounds were common, we found differences between the parts tested. However, we examined the differences in the same partial extracts of the tested *Cistus* species.

### 3.2. Total Phenolic and Flavonoid Contents

The objective of this study was to determine the total phenolic content (TPC) and total flavonoid content (TFC) of methanolic extracts of various components (leaves, roots, and twigs) of two *Cistus* species, specifically *C. monspeliasis* and *C. parviflorus*. Phenolics and flavonoids are plant metabolites that have undergone thorough examination due to their antioxidant, anti-inflammatory, and antibacterial characteristics. The unique characteristics of these substances make them extremely beneficial for a wide range of pharmacological and nutraceutical uses [50,51,52,53]. The measurements were expressed in milligrams of gallic acid equivalents per gram (mg GAE/g) for phenolics and milligrams of rutin equivalents per gram (mg RE/g) for flavonoids. The mean values plus or minus standard deviations were computed based on three parallel measurements. All the results are presented in Table 3.

The investigation on *C. monspeliasis* revealed that the roots had the highest total phenolic content (TPC), measuring 103.35 mg GAE/g. The twigs exhibited the second-greatest total phenolic content (TPC) with a value of 98.59 mg Gallic Acid Equivalent (GAE) per gram, whilst the leaves displayed the lowest TPC with a value of 52.07 mg GAE/g. The leaves had the highest total flavonoid content (TFC) at 56.98 mg RE/g, whereas the twigs and roots had much lower values of 9.29 mg RE/g and 2.43 mg RE/g, respectively. The leaves of *C. parviflorus* were found to have a high TPC (total phenolic content) of 94.44 mg GAE/g. Nevertheless, it exhibited a minor decrease in comparison to the TPC in the roots (101.13 mg GAE/g) and twigs (96.99 mg GAE/g). Based on our investigation, the leaves showed the greatest total flavonoid content (TFC) with a value of 43.19 mg RE/g. Comparatively, the twigs exhibited a total flavonoid content (TFC) of 5.86 mg RE/g, but the roots displayed the lowest TFC of 1.91 mg RE/g (Table 3).

In a separate investigation conducted by Haida et al. [18], it was discovered that extracts of *C. monspeliensis* contain a significant amount of phenolic chemicals, specifically flavonoids (69.81 ± 0.22 mg EQ/g DM), hydrolysable tannins (61.86 ± 0.89 mg ETA/g DM), and condensed tannins (70.05 ± 1.61 mg EC/g DM). The results of our investigation indicate that the roots of the plant have a greater total phenolic content (TPC) of 103.35 ± 0.54 mg GAE/g compared to the leaves (52.07 ± 0.21 mg GAE/g) and twigs (98.59 ± 0.49 mg GAE/g). This suggests that different portions of the plant contain varied concentrations of these compounds [18]. According to Tawaha et al. [54], a total phenolic content greater than 20 mg EGA/g dry weight is classified as being very high. Thus, all samples of *C. monspeliensis* analysed in this study can be considered an outstanding reservoir of phenolic chemicals. In a prior investigation, the ethanolic extract of *C. monspeliensis* from Ouazzane, a city in northern Morocco, exhibited a total polyphenol content of 79.19 ± 2.42 mg EGA/g DM in a polyphenols test, along with a flavonoid content of 19.43 mg EQ/g [55].

### 3.3. Antioxidant Activities

The current research was designed to investigate the antioxidant activities of methanolic extracts of the various parts (twigs, roots, and leaves) of two *Cistus* species (*C. monspeliasis* and *C. parviflorus*) by different assays: including DPPH, ABTS, CUPRAC, FRAP, Chelating, and PBD. The results presented in Table 4 indicate a strong association between the overall phenolic content (TPC) and the antioxidant properties of the different portions of the plant. Phenolics are recognised for their strong antioxidant characteristics, which help to eliminate free radicals and decrease oxidative stress [56,57].

The DPPH assay quantifies the capacity to eliminate or neutralise free radicals. The roots of *C. monspeliasis* have the most significant activity, measuring 651.30 mg TE/g. The twigs follow closely with a measurement of 564.54 mg TE/g, while the leaves show a lower activity of 74.76 mg TE/g. Similarly, the roots of *C. parviflorus* exhibit a significant level of activity, measuring 648.83 mg TE/g. This is followed by the twigs, which show a somewhat lower activity of 566.34 mg TE/g, and the leaves, which have a measured activity of 532.07 mg TE/g (Table 4). This implies that the roots with the highest total phenolic content (TPC) exhibited greater efficacy in scavenging free radicals.

We additionally assessed the capacity of scavenging free radicals by employing the ABTS assay. The roots of *C. monspeliasis* exhibit a high activity level, with a value of 851.53 mg TE/g. The twigs also reveal high activity, with a value of 784.89 mg TE/g. However, the leaves of *C. monspeliasis* show significantly lower values, with a measurement of 85.62 mg TE/g. The roots of *C. parviflorus* have a high activity level of 854.90 mg TE/g, while the twigs have a somewhat lower activity level of 783.65 mg TE/g. The leaves of *C. parviflorus* perform better than those of *C. monspeliasis*, with an activity level of 681.94 mg TE/g (Table 4). This further corroborates the conclusion that the roots possess exceptional antioxidant properties.

The FRAP assay measures the ability of a substance to reduce ferric ions and act as an antioxidant. The roots of *C. parviflorus* have a high activity level of 541.41 mg TE/g, while the twigs have a somewhat lower activity level of 430.68 mg TE/g. On the other hand, the leaves of *C. parviflorus* have a lower activity level of 339.03 mg TE/g. In addition to twigs (384.99 mg TE/g) and leaves (89.38 mg TE/g), the roots of *C. monspeliasis* (481.89 mg TE/g) also exhibit significant activity (Table 4). The constant trend observed in the roots consistently displaying the highest activity level indicates that phenolic chemicals are the primary factors responsible for their strong reducing ability. The extracts were assessed by Haida, Sara, et al. utilising the FRAP and DPPH tests. They discovered that the butanolic and water/acetone extracts exhibited remarkable IC50 values of 0.099 and 0.079 mg/mL, respectively. These outcomes validate *C. monspeliensis*’s strong antioxidant capacity, confirming our conclusions and emphasising the plant’s potential in medical applications [18].

The CUPRAC assay quantifies the ability of a substance to reduce cupric ions and so determine its antioxidant capability. Results are summarised in Table 4. The roots (938.11 mg TE/g), twigs (710.45 mg TE/g), and leaves (609.68 mg TE/g) of *C. parviflorus* exhibit the highest levels of activity. The roots of *C. monspeliasis* exhibit the highest activity with a value of 843.01 mg TE/g, followed by the twigs with a value of 684.74 mg TE/g, and the leaves with a value of 123.23 mg TE/g. This strengthens the considerable antioxidant capacity of the roots, attributed to their elevated total phenolic content (TPC). Chelating tests are utilised to assess the metal ion binding capacity (mg EDTAE/g) of various plant components [58]. The roots exhibited the highest chelating activity, measuring 15.01 mg EDTAE/g. The twigs showed a lower chelating activity of 5.03 mg EDTAE/g, while the leaves had the lowest chelating activity of 4.49 mg EDTAE/g. The leaves of *C. monspeliasis* exhibit a moderate activity level, with the twigs and roots having lower values of EDTAE content. These findings suggest that the leaves possess notable metal chelating abilities, perhaps due to their abundant flavonoid content.

**Figure 2:**
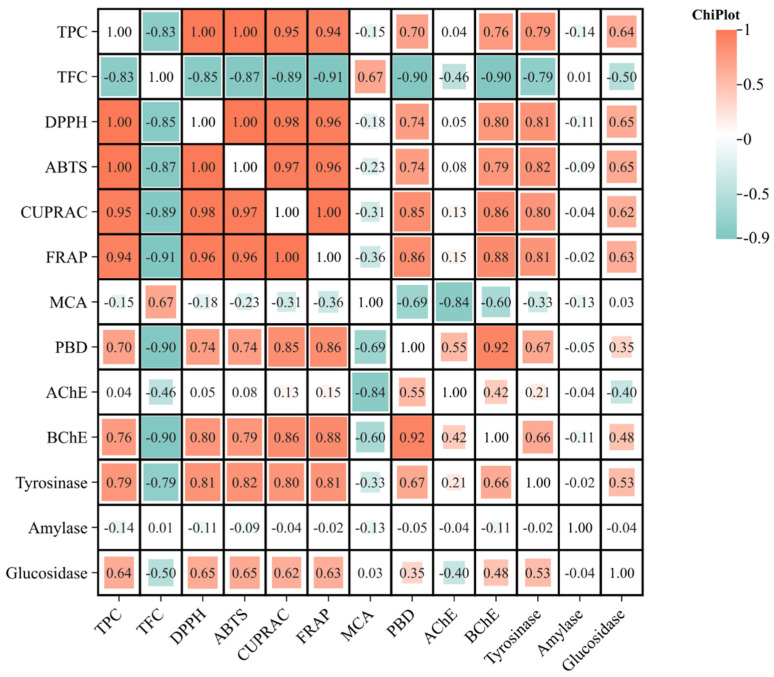
Pearson correlation between phenolic components and antioxidant and enzyme inhibitory effects (*p* < 0.05). ABTS, 2,2-azino-bis(3-ethylbenzothiazoline) 6-sulfonic acid; AChE, acetylcholinesterase; BChE, butyrylcholinesterase; CUPRAC, cupric ion-reducing antioxidant capacity; DPPH, 1,1-diphenyl-2-picrylhydrazyl; FRAP, ferric ion-reducing antioxidant power; MCA, metal chelating activity; PBD, Phosphomolybdenum. TPC, Total phenolic content; TFC, Total flavonoid content. (R > 0.7 indicates strong correlation between phenolic components and biolocal activities).

The PBD assay quantifies the antioxidative potential of phosphomolybdenum [59]. The roots (5.02 mmol TE/g), twigs (3.92 mmol TE/g), and leaves (3.05 mmol TE/g) of *C. parviflorus* exhibit the highest levels of activity. The roots of *C. monspeliasis* exhibit a high activity level of 4.79 mmol TE/g, whereas the twigs and leaves show slightly lower activity levels of 4.13 mmol TE/g and 2.90 mmol TE/g, respectively (Table 4). This pattern once again highlights the notable antioxidant ability of the roots, which is most likely due to their elevated total phenolic content (TPC). The findings demonstrated a distinct association between the antioxidant properties of the different components of the plant and their overall levels of phenolic and flavonoid compounds (Figure 2). Despite having the highest total flavonoid content (TFC), the leaves of *C. monspeliasis* exhibited inferior antioxidant activity in most tests, except the chelating assay. This suggests that there are other phenolic molecules, in addition to flavonoids, that are essential for the antioxidant properties of these plant components.

The findings emphasise the variation in antioxidant activity across different plant components and propose the selective utilisation of specific sections based on their phytochemical composition. Acquiring this understanding is crucial for creating health-related products using these *Cistus* species and harnessing their powerful antioxidant qualities for pharmaceutical and nutraceutical purposes.

### 3.4. Enzyme Inhibitory Activities

Acetylcholinesterase (AChE) and butyrylcholinesterase (BChE) are vital enzymes involved in the progression of Alzheimer’s disease (AD). Cholinesterase inhibitors have consistently shown effectiveness in treating both mild and severe types of Alzheimer’s disease (AD). Galanthamine, a chemical derived from plants, is the most recent anticholinesterase medication among the inhibitors mentioned [60]. Moreover, studies have shown a connection between the advancement of Alzheimer’s disease (AD) and the imbalance of iron regulation and oxidative stress [61]. Many previous studies suggest that the *Cistus* plant species contain several compounds that have a great potential to inhibit these important enzymes [62,63]. Hence, the objective of this study is to investigate the cholinesterase inhibitory capabilities of methanolic extracts obtained from the different parts of *C. monspeliensis* and *C. parviflorus*.

The inhibition of acetylcholinesterase (AChE) in *C. monspeliensis* varied, with the lowest activity observed in the leaves (2.54 ± 0.04 mg GALAE/g) and the highest in the roots (2.58 ± 0.02 mg GALAE/g). When compared, *C. parviflorus* showed a wider range of AChE inhibition. The leaves exhibited 2.44 ± 0.03 mg GALAE/g, while the roots showed 2.53 ± 0.01 mg GALAE/g (Table 5). This suggests that *C. monspeliensis* had a little better overall AChE inhibition. *C. monspeliensis* displayed the least activity of butyrylcholinesterase (BChE) in its leaves (3.69 ± 0.69 mg GALAE/g) and the highest activity in its roots (11.37 ± 1.93 mg GALAE/g). In contrast, *C. parviflorus* exhibited BChE inhibition ranging from 5.38 ± 0.87 mg GALAE/g in the leaves to 10.90 ± 0.62 mg GALAE/g in the roots. These findings indicate that *C. monspeliensis* had greater BChE inhibition, particularly in the roots. On the other hand, AChE inhibitory action was not observed in Loizzo et al.’s [63] investigations on *C. monspeliensis* essential oil (EO), and BChE inhibition was marginally observed. The reason behind this disparity was the restricted existence of monoterpenes with AChE inhibitory characteristics, like α-pinene, β-pinene, and α-terpinene.

Cistus extracts were evaluated for their tyrosinase inhibitory activity. Tyrosinase is an enzyme that contains copper and is responsible for catalysing the conversion of L-tyrosine to L-DOPA and subsequently to dopaquinone. This enzymatic process is a crucial step in the creation of melanin. Tyrosinase is a key target for skin-lightening cosmetics that attempt to reduce hyperpigmentation because it plays a vital role in melanin formation [64]. Hyperpigmentation, which is the localised buildup of melanin pigment, is frequently induced by excessive ultraviolet (UV) exposure and is a major concern for cosmetic products aimed at safeguarding the skin [65]. *C. monspeliensis* exhibited tyrosinase (Tyr) inhibitory activity ranging from 63.09 ± 4.45 mg KAE/g in the leaves to 70.87 ± 0.16 mg KAE/g in the roots. However, *C. parviflorus* exhibited tyrosinase inhibition. The leaves showed a value of 68.03 ± 1.61 mg KAE/g, while the twigs showed a maximum activity of 71.46 ± 1.38 mg KAE/g. This suggests that *C. parviflorus* has a stronger overall tyrosinase inhibition (Table 5).

Diabetes mellitus is a major chronic metabolic disease that is associated with significant health problems and a high death rate [66]. One established risk factor for the development of diabetes is excessive postprandial glucose excursions [67]. A fascinating strategy to control the deviation is to suppress the activity of digestive enzymes that produce glucose, such as α-amylase and α-glucosidase [68]. The study revealed that extracts from *C. monspeliensis* and *C. parviflorus* displayed notable inhibitory activity against α-amylase and α-glucosidase enzymes (Table 5). Regarding α-amylase inhibition, *C. monspeliensis* exhibited activity levels ranging from 0.58 ± 0.02 mmol ACAE/g in the roots to 0.63 ± 0.03 mmol ACAE/g in the leaves. The range of *C. parviflorus* varied slightly, with the leaves measuring 0.61 ± 0.02 mmol ACAE/g and the roots measuring 0.65 ± 0.04 mmol ACAE/g. A recent study examined the inhibitory activity of *C. monspeliensis* extracts on α-amylase. The results showed that the extracts displayed a range of IC50 values, indicating a significant potential for inhibition [69]. Our results are expressed in different units (mmol ACAE/g), but the lower IC50 values from the other study indicate that *C. monspeliensis* extracts have a significant inhibitory effect on α-amylase, which may be like the effective range observed in our study. Concerning α-glucosidase inhibition, our findings revealed that *C. parviflorus* exhibited inhibition from 1.07 ± 0.03 mmol ACAE/g in the leaves to 1.09 ± 0.03 mmol ACAE/g in the twigs, while *C. monspeliensis* demonstrated activity from 0.94 ± 0.12 mmol ACAE/g in the leaves to 1.03 ± 0.04 mmol ACAE/g in the twigs. With IC50 values ranging from 0.95 ± 0.14 to 14.58 ± 1.26 µg/mL, *C. monspeliensis* extracts showed a substantial inhibitory impact on α-glucosidase in the other investigation. Our results, which also demonstrate effective inhibition, are consistent with these lower IC50 values, which reveal a significant inhibition and imply that *C. monspeliensis* extracts have a strong inhibitory impact on α-glucosidase [69]. Our findings regarding the inhibition of α-amylase and α-glucosidase align with the conclusions of previous research indicating the potent inhibitory effect of extracts from other cistus species [70,71,72]. This comparison demonstrates how these extracts may effectively suppress certain enzymes to manage diabetes.

### 3.5. Antimicrobial Activity

The MIC values of extracts from two *Cistus* species against bacteria, yeasts, and dermatophytes are presented in Table 6, Table 7 and Table 8. Each *Cistus*’s extracts demonstrated antimicrobial activity within a concentration range of 1.562 to 200 μg/mL. Notably, *Escherichia coli* (ATCC 10536) and *Pseudomonas aeruginosa* (ATCC 15442) were highly susceptible to the twig extract of *C. monspeliensis* and the leaves extract of *C. parviflorus*, with MIC values of 3.125–6.355 μg/mL (GM, 3.96 μg/mL) and 6.25–12.5 µg/mL (GM, 9.92 µg/mL), respectively. In contrast, *Bacillus subtilis* (PeruMycA 6) and *Salmonella typhi* (PeruMyc 7) exhibited minimal sensitivity to most of the tested extracts. Generally, Gram-negative bacterial strains, such as *S. typhi* (PeruMycA7), demonstrated less susceptibility to plant extracts compared to their Gram-positive strains. The observed differences in antibacterial effectiveness of *Cistus* extracts against Gram-negative and Gram-positive bacteria can be attributed to their distinct cell surfaces characteristics, as highlighted by Tamboli and Lee (2013). Gram-negative bacteria possess an additional outer layer that protects them from hostile environments by excluding toxic molecules while allowing the exchange of essential nutrients and materials [73].

Similar results were observed with *Fuscoporia torulosa* and *Pleurotus* mushroom species [74,75,76]. Remarkably, only the twig extracts of *C. parviflorus* significantly inhibited *Candida tropicalis* (YEPGA 6184), with MIC values ranging from 12.5 to 25 μg/mL (GM 15.75 μg/mL). Furthermore, the extracts significantly inhibited dermatophyte growth, with *Trichophyton tonsurans* (CCF 4834) and *Arthroderma insingulare* (CCF 5417) being the most responsive, showing MIC values between 31.49 and 62.99 μg/mL (Table 8). The MIC values for ciprofloxacin, fluconazole, and griseofulvin against *C. parapsilosis* (ATCC 22019) and *C. krusei* (ATCC 6258) adhered to the benchmarks set by CLSI, 2008b.

According to Seil and Webster [77], MIC values under 100 μg/mL generally indicate strong antimicrobial activity. In this study, some MIC values were lower than 100 μg/mL, suggesting that the investigated extracts possessed moderate antimicrobial properties. Comparing bioactivity outcomes across different studies is challenging due to variations in extraction methods, test organisms, and experimental systems utilised [78].

### 3.6. Cytotoxic Effects

Cytotoxicity experiments were conducted to assess the effects of several extracts derived from *C. monspeliensis* and *C. parviflorus* on RAW, HepG2, and S17 cell lines at a single concentration of 100 µg/mL. The results are expressed as a percentage of cellular viability relative to a control using 0.5% DMSO and summarised in Table 9.

According to the ISO 10993-5 [79] criteria for in vitro toxicity assessment, cell viability greater than 80% indicates non-cytotoxicity, 60–80% suggests weak cytotoxicity, 40–60% represents moderate cytotoxicity, and less than 40% indicates strong cytotoxicity [79]. Using this standard, the cytotoxicity profile of *Cistus* extracts can be interpreted accordingly.

The extracts exhibited varying degrees of cytotoxicity across the different cell lines. In HepG2 cell line, the leaf extracts of *C. monspeliensis* showed moderate cytotoxicity, with a cell viability of 48.8%, while the root and twig extracts exhibited no cytotoxicity, maintaining cell viabilities above 97%. Similarly, for *C. parviflorus*, the root extracts exhibited weak to moderate cytotoxicity (60.3%), whereas the twig extracts showed no cytotoxicity, with a cell viability of 82.6%. For RAW cells, the leaf extracts of both species displayed moderate cytotoxicity, with cellular viabilities ranging from 58.1% to 60.1%. In contrast, the root extracts of both species showed weak cytotoxicity, with viabilities between 70.9% and 71.4%, according to ISO 10993-5 standards [79]. Notably, all extracts demonstrated high cell viability in S17 cells (98.3% to 112%), indicating no cytotoxicity and suggesting potential biocompatibility.

Medicinal plants offer a rich source of bioactive compounds with therapeutic potential, but their application is often limited by concerns over possible toxic effects, which could lead to health risks. Therefore, assessing the safety and biocompatibility of these extracts is essential to validate their potential for therapeutic use [80]. While previous studies have shown that other *Cistus* species, such as *C. incanus*, *C. villosus*, and *C. salviifolius*, exhibit cytotoxic effects against various cancer cells, including prostate, breast, and melanoma cells [81,82,83], our findings contrast with these, as we observed generally low cytotoxicity in our extracts. The variation in cytotoxicity profiles underscores the importance of species-specific investigations into medicinal plant extracts and highlights the need for thorough safety evaluations. Our results indicate that the extracts of *C. monspeliensis* and *C. parviflorus* are generally safe, exhibiting no toxicity in S17 cells and weak toxicity in most plant organs when tested on RAW and HepG2 cells. This supports their potential for therapeutic use. However, some extracts have demonstrated moderate toxicity to RAW and HepG2 cell lines. Fractionation of these extracts may help isolate and mitigate the toxic components, thereby enhancing their overall safety profile. Nonetheless, further research is needed to confirm their safety and to investigate the specific mechanisms underlying their therapeutic effects.

## 4. Conclusions

*Cistus* species have historically been employed as treatments in various traditional folk medicines across diverse populations globally. The current research was designed to investigate the therapeutic potential of two cistus species from Turkey flora. According to the findings, both the plant species contain a significant number of total phenolic contents and flavonoids and demonstrate a significant antioxidant potential across all the evaluated assays. Moreover, *C. monspeliensis* showed a greater ability to inhibit acetylcholinesterase and butyrylcholinesterase, while *C. parviflorus* displayed better inhibition of tyrosinase, α-amylase, and α-glucosidase. Additionally, the findings also suggest that both species possess antimicrobial and low cytotoxic towards mammalian cell lines. This comparative investigation highlights the potential of both plants as reservoirs of bioactive chemicals for the development of natural treatments for Alzheimer’s disease, skin problems, and diabetes.

## Figures and Tables

**Table 1:** Comparison of the identified compounds of the extracts from *Cistus parviflorus*.

Compounds	Leaves	Twigs	Roots
Quinic acid	+	+	+
Citric acid	+	+	+
Arbutin	+	+	+
Prodelphinidin B isomer 1	-	+	+
Gallic acid (3,4,5-Trihydroxybenzoic acid)	+	+	+
Prodelphinidin B isomer 2	-	+	+
Protocatechuic acid (3,4-Dihydroxybenzoic acid)	+	+	+
Prodelphinidin B isomer 3	-	+	+
Gallocatechin	+	+	+
Prodelphinidin B isomer 4	-	+	+
Procyanidin B isomer 1	-	+	+
Punicalagin isomer	+	-	-
Flavogallonic acid dilactone or isomer	+	+	-
Punicalagin	+	-	-
Prodelphinidin B isomer 5	-	+	+
Procyanidin B isomer 2	-	+	+
Esculin (Esculetin-6-O-glucoside)	+	+	+
Unidentified hydroxybenzoic acid derivative 1	+	+	+
Catechin	+	+	+
Epigallocatechin	+	+	+
Magnolioside (Isoscopoletin-6-O-glucoside)	-	+	+
Scopolin (Scopoletin-7-O-glucoside)	-	+	+
Caffeic acid-O-glucoside	+	+	-
Caffeic acid	+	+	+
Unidentified hydroxybenzoic acid derivative 2	+	+	+
Naringenin-6,8-di-C-glucoside	+	+	-
Epigallocatechin-3-O-gallate (Teatannin II)	+	+	+
Epicatechin	+	+	+
Isoscopoletin (6-Hydroxy-7-methoxycoumarin)	+	+	+
p-Coumaric acid	+	+	+
Vicenin-2 (Apigenin-6,8-di-C-glucoside)	+	+	-
Scopoletin (7-Hydroxy-6-methoxycoumarin)	+	+	+
Methyl flavogallonate	+	+	-
Ellagic acid-4-O-glucoside	+	+	-
Quercetin-O-dirhamnosylhexoside	+	+	-
Myricetin-3-O-glucoside (Isomyricitrin)	+	+	+
Quercetin-O-galloylhexoside	+	-	-
Kaempferol-O-dirhamnosylhexoside	+	+	-
Myricetin-3-O-pentoside	+	+	-
3-O-Methylellagic acid-4′-O-glucoside	-	+	-
Myricitrin (Myricetin-3-O-rhamnoside)	+	+	+
Kaempferol-3-O-neohesperidoside	+	+	-
Myricetin-O-malonylhexoside	+	-	-
Hyperoside (Quercetin-3-O-galactoside)	+	+	+
Ellagic acid-O-pentoside	+	+	+
Isoquercitrin (Quercetin-3-O-glucoside)	+	+	-
Rutin (Quercetin-3-O-rutinoside)	+	+	-
Eschweilenol C (Ellagic acid-4-O-rhamnoside)	-	+	-
Avicularin (Quercetin-3-O-arabinofuranoside)	+	+	-
Ellagic acid	+	+	+
Kaempferol-7-O-glucoside	+	+	-
Quercetin-O-malonylhexoside	+	-	-
Guaijaverin (Quercetin-3-O-arabinopyranoside)	+	-	-
Dimethoxy-trihydroxyflavone-O-hexoside	+	+	+
Astragalin (Kaempferol-3-O-glucoside)	+	+	+
Kaempferol-3-O-rutinoside (Nicotiflorin)	+	+	-
Ducheside A (3-O-Methylellagic acid-4′-O-xyloside)	-	+	-
3-O-Methylellagic acid-4′-O-rhamnoside	-	+	+
3-O-Methylellagic acid	-	+	-
Isorhamnetin-3-O-rutinoside (Narcissin)	+	-	-
Kaempferol-O-malonylhexoside	+	-	-
Pinobanksin (3,5,7-Trihydroxyflavanone)	+	+	-
Naringenin (4′,5,7-Trihydroxyflavanone)	+	+	+
Quercetin (3,3′,4′,5,7-Pentahydroxyflavone)	+	+	+
Kaempferol-3-O-[rhamnosyl-(1→2)-(6″-O-trans-p-coumaroyl)]glucoside	+	+	-
Di-O-methylellagic acid	-	+	-
Tiliroside (6″-O-trans-p-Coumaroylastragalin)	+	+	+
Quercetin-3-O-methyl ether	+	+	-
3″-O-trans-p-Coumaroylastragalin	+	+	+
Axillarin (3,6-Dimethoxy-3′,4′,5,7-tetrahydroxyflavone)	+	+	-
Kaempferol (3,4′,5,7-Tetrahydroxyflavone)	+	+	-
Isorhamnetin (3′-Methoxy-3,4′,5,7-tetrahydroxyflavone)	+	+	-
Apigenin (4′,5,7-Trihydroxyflavone)	+	+	-
Chrysoeriol (3′-Methoxy-4′,5,7-trihydroxyflavone)	+	+	-
Isokaempferide (3-Methoxy-4′,5,7-trihydroxyflavone)	+	+	-
3,8-Dimethoxy-4′,5,7-trihydroxyflavone	+	+	+
Rhamnetin (7-Methoxy-3,3′,4′,5-tetrahydroxyflavone)	+	+	-
Jaceidin (4′,5,7-Trihydroxy-3,3′,6-trimethoxyflavone)	+	-	-
Pinocembrin (5,7-Dihydroxyflavanone)	+	+	+
3,6-Dimethoxy-4′,5,7-trihydroxyflavone	+	+	+
4′,5,7-Trihydroxy-3,3′,8-trimethoxyflavone (Gossypetin-3,3′,8-trimethyl ether)	+	+	-
Kaempferol-3-O-(3,6-di-p-coumaroylglucoside)	+	+	-
Dihydroxy-trimethoxy(iso)flavone isomer 1	+	-	-
Dihydroxy-trimethoxy(iso)flavone isomer 2	+	+	-
Dihydroxy-tetramethoxy(iso)flavone	+	+	-
5,7-Dihydroxy-3,4′,8-trimethoxyflavone (Herbacetin-3,4′,8-trimethyl ether)	+	+	+
5,7-Dihydroxy-3,3′,4′,8-tetramethoxyflavone (Gossypetin-3,3′,4′,8-tetramethyl ether)	+	+	+
Flindulatin (5-Hydroxy-3,4′,7,8-tetramethoxyflavone)	+	+	+
Kaempferol-3,4′,7-trimethyl ether (5-Hydroxy-3,4′,7-trimethoxyflavone)	+	+	-
Pheophytin A	+	-	-

+: present; -: absent.

**Table 2:** Comparison of the identified compounds of the extracts from *C. monspeliasis*.

Compounds	Leaves	Twigs	Roots
Quinic acid	+	+	+
Malic acid	+	+	+
Citric acid	+	+	+
Gallic acid (3,4,5-Trihydroxybenzoic acid)	+	+	+
Gentisic acid (2,5-Dihydroxybenzoic acid)	+	+	+
Gallocatechin	+	+	+
Gentisic acid-O-glucoside	+	+	+
Procyanidin B isomer 1	-	+	+
Flavogallonic acid dilactone or isomer	+	+	-
Uralenneoside	+	+	+
Procyanidin B isomer 2	-	+	+
Esculin (Esculetin-6-O-glucoside)	+	+	-
Unidentified hydroxybenzoic acid derivative 1	-	-	+
Catechin	+	+	+
Epigallocatechin	+	+	+
Procyanidin B isomer 3	-	+	+
Magnolioside (Isoscopoletin-6-O-glucoside)	-	+	+
Scopolin (Scopoletin-7-O-glucoside)	+	+	+
Esculetin (6,7-Dihydroxycoumarin)	+	+	
Procyanidin B isomer 4	-	+	+
Fraxetin-O-glucoside	-	+	-
Monspelioside (1-(3,5-Dihydroxy-2-methylphenyl)ethanone-5-O-glucoside)	+	+	+
Unidentified hydroxybenzoic acid derivative 2	-	-	+
Naringenin-6,8-di-C-glucoside	-	+	+
Epicatechin	+	+	+
Fraxetin (7,8-Dihydroxy-6-methoxycoumarin)	+	+	-
Isoscopoletin (6-Hydroxy-7-methoxycoumarin)	-	+	+
p-Coumaric acid	+	+	+
Vicenin-2 (Apigenin-6,8-di-C-glucoside)	+	+	+
Scopoletin (7-Hydroxy-6-methoxycoumarin)	+	+	+
Taxifolin (Dihydroquercetin)	+	+	-
Ellagic acid-4-O-glucoside	+	+	-
Sinapic acid	+	-	-
Dimethoxy-hydroxycoumarin	+	+	+
Myricetin-3-O-glucoside (Isomyricitrin)	+	+	+
Scoparone (6,7-Dimethoxycoumarin)	+	-	-
Myricetin-O-pentoside isomer 1	+	+	-
Myricetin-O-pentoside isomer 2	+	+	-
Dihydrokaempferol (3,4′,5,7-Tetrahydroxyflavanone)	+	+	-
Myricitrin (Myricetin-3-O-rhamnoside)	+	+	-
Quercetin-O-pentosylhexoside	+	-	-
Myricetin-O-pentoside isomer 3	+	+	-
Hyperoside (Quercetin-3-O-galactoside)	+	+	+
Isoquercitrin (Quercetin-3-O-glucoside)	-	+	+
Trimethoxycoumarin	+	-	-
Ellagic acid-O-pentoside	+	+	-
Rutin (Quercetin-3-O-rutinoside)	+	-	-
Eschweilenol A or isomer	+	-	-
Ellagic acid	+	+	+
Kaempferol-7-O-glucoside	+	+	-
Myricetin (3,3′,4′,5,5′,7-Hexahydroxyflavone)	+	+	-
Quercitrin (Quercetin-3-O-rhamnoside)	+	+	+
3-O-Methylellagic acid-4′-O-rhamnoside	-	+	+
Pinobanksin (3,5,7-Trihydroxyflavanone)	+	+	-
Quercetin-O-coumaroylhexoside	+	+	-
Naringenin (4′,5,7-Trihydroxyflavanone)	-	+	-
Quercetin (3,3′,4′,5,7-Pentahydroxyflavone)	+	+	+
Trihydroxy-trimethoxy(iso)flavone-O-hexoside	+	+	-
3,4′-Di-O-methylellagic acid	-	+	-
Luteolin (3′,4′,5,7-Tetrahydroxyflavone)	+	+	-
3,3′-Di-O-methylellagic acid	-	-	+
3,4′-Di-O-methylellagic acid	-	+	-
Tiliroside (6″-O-trans-p-Coumaroylastragalin)	+	+	-
Quercetin-3-O-methyl ether	+	+	+
Dimethoxy-tetrahydroxy(iso)flavone	+	+	-
Kaempferol (3,4′,5,7-Tetrahydroxyflavone)	+	+	-
Isorhamnetin-7-O-rhamnoside	+	-	-
Isorhamnetin (3′-Methoxy-3,4′,5,7-tetrahydroxyflavone)	+	+	+
Apigenin (4′,5,7-Trihydroxyflavone)	+	+	+
Chrysoeriol (3′-Methoxy-4′,5,7-trihydroxyflavone)	+	+	+
Isokaempferide (3-Methoxy-4′,5,7-trihydroxyflavone)	+	+	+
Dimethoxy-trihydroxy(iso)flavone	+	+	+
Rhamnetin (7-Methoxy-3,3′,4′,5-tetrahydroxyflavone)	+	+	+
Trihydroxy-trimethoxy(iso)flavone isomer 1	+	+	+
Malyngic acid (9,12,13-Trihydroxy-10E,15Z-octadecadienoic acid)	-	-	+
Pinocembrin (5,7-Dihydroxyflavanone)	+	+	+
Luteolin-7-O-methyl ether (7-Methoxy-3′,4′,5-trihydroxyflavone)	+	+	+
Trihydroxy-trimethoxy(iso)flavone isomer 2	+	+	+
Pinellic acid (9,12,13-Trihydroxy-10E-octadecenoic acid)	-	-	+
Dihydroxy-trimethoxy(iso)flavone isomer 1	+	+	-
Dihydroxy(iso)flavone	+	+	-
Methoxy-trihydroxy(iso)flavone isomer 1	+	+	-
Acacetin (5,7-Dihydroxy-4′-methoxyflavone)	+	+	-
Methoxy-trihydroxy(iso)flavone isomer 2	+	+	-
Genkwanin (4′,5-Dihydroxy-7-methoxyflavone)	+	+	-
Kumatakenin (4′,5-Dihydroxy-3,7-dimethoxyflavone)	+	+	-
Dihydroxy-trimethoxy(iso)flavone isomer 2	+	+	-
Ermanin (5,7-Dihydroxy-3,4′-dimethoxyflavone)	+	+	+
Dihydroxy-trimethoxy(iso)flavone isomer 3	+	+	-
Myricetin-3,3′,4′,7-tetramethyl ether (5,5′-Dihydroxy-3,3′,4′,7-tetramethoxyflavone)	+	+	+
Vitexilactone or isomer	+	+	+
Emodin	-	-	+
Hydroxy-tetramethoxy(iso)flavone	+	+	-
Hydroxy-methoxy(iso)flavone	+	+	-
Apigenin-4′,7-dimethyl ether (4′,7-Dimethoxy-5-hydroxyflavone)	+	+	-
Kaempferol-3,4′,7-trimethyl ether (5-Hydroxy-3,4′,7-trimethoxyflavone)	+	+	-
18-Hydroxy-cis-clerodan-3-ene-15-oic acid or 15-Hydroxy-cis-clerodan-3-ene-18-oic acid	+	+	+
18-Hydroxy-cis-clerodan-3-ene-15-oic acid or 15-Hydroxy-cis-clerodan-3-ene-18-oic acid	-	-	+
18-Hydroxy-cis-clerodan-3-ene-15-oic acid or 15-Hydroxy-cis-clerodan-3-ene-18-oic acid	-	-	+
Cistadiol (15,18-Dihydroxy-cis-clerodan-3-ene)	+	+	-
18-Acetoxy-cis-clerodan-3-ene-15-oic acid or 15-Acetoxy-cis-clerodan-3-ene-18-oic acid	+	+	-
8-Hydroxylabdan-15-oic acid	+	+	+
Pheophytin A	+	+	-

+: present; -: absent.

**Table 3:** Total phenolic and flavonoid contents in the tested extracts.

Species	Parts	TPC (mg GAE/g)	TFC (mg RE/g)
*Cistus monspeliasis*	Leaves	52.07 ± 0.21 ^d^	56.98 ± 0.26 ^a^
	Roots	103.35 ± 0.54 ^a^	2.43 ± 0.15 ^e^
	Twigs	98.59 ± 0.49 ^b^	9.29 ± 0.23 ^c^
*Cistus parviflorus*	Leaves	94.44 ± 0.15 ^c^	43.19 ± 0.44 ^b^
	Roots	101.13 ± 1.26 ^a^	1.91 ± 0.08 ^e^
	Twigs	96.99 ± 1.43 ^b^	5.86 ± 0.04 ^d^

Values are reported as mean ± SD of three parallel measurements. GAE: Gallic acid equivalents; RE: Rutin equivalents. Different letters indicate significant differences between the tested extracts (*p* < 0.05).

**Table 4:** Antioxidant properties of the tested extracts.

Species	Parts	DPPH (mg TE/g)	ABTS (mg TE/g)	CUPRAC (mg TE/g)	FRAP (mg TE/g)	Chelating (mg EDTAE/g)	PBD (mmol TE/g)
*Cistus monspeliasis*	Leaves	74.76 ± 0.15 ^d^	85.62 ± 0.04 ^d^	123.23 ± 4.61 ^f^	89.38 ± 1.33 ^f^	7.23 ± 0.71 ^b^	2.90 ± 0.02 ^c^
	Roots	651.30 ± 3.11 ^a^	851.53 ± 0.59 ^a^	843.01 ± 5.00 ^b^	481.89 ± 4.31 ^b^	4.58 ± 0.10 ^c^	4.79 ± 0.07 ^a^
	Twigs	564.54 ± 3.45 ^b^	784.89 ± 0.94 ^b^	684.74 ± 9.83 ^d^	384.99 ± 4.35 ^d^	5.30 ± 0.36 ^c^	4.13 ± 0.07 ^b^
*Cistus parviflorus*	Leaves	532.07 ± 5.86 ^c^	681.94 ± 13.92 ^c^	609.68 ± 10.24 ^e^	339.03 ± 7.15 ^e^	15.01 ± 0.25 ^a^	3.05 ± 0.10 ^c^
	Roots	648.83 ± 0.97 ^a^	854.90 ± 0.62 ^a^	938.11 ± 9.57 ^a^	541.41 ± 2.58 ^a^	4.49 ± 0.31 ^c^	5.02 ± 0.33 ^a^
	Twigs	566.34 ± 1.95 ^b^	783.65 ± 6.50 ^b^	710.45 ± 8.78 ^c^	430.68 ± 7.41 ^c^	5.03 ± 0.22 ^c^	3.92 ± 0.13 ^b^

Values are reported as mean ± SD of three parallel measurements. PBD: Phosphomolybdenum; TE: Trolox Equivalent; EDTAE: EDTA equivalent. Different letters indicate significant differences between the tested extracts (*p* < 0.05).

**Table 5:** Enzyme inhibitory properties of the tested extracts.

Species	Parts	AChE (mg GALAE/g)	BChE (mg GALAE/g)	Tyrosinase (mg KAE/g)	Amylase (mmol ACAE/g)	Glucosidase (mmol ACAE/g)
*Cistus monspeliasis*	Leaves	2.54 ± 0.04 ^a^	3.69 ± 0.69 ^d^	63.09 ± 4.45 ^b^	0.63 ± 0.03 ^a^	0.94 ± 0.12 ^b^
	Roots	2.58 ± 0.02 ^a^	11.37 ± 1.93 ^a^	70.87 ± 0.16 ^a^	0.58 ± 0.02 ^a^	1.03 ± 0.03 ^ab^
	Twigs	2.56 ± 0.01 ^a^	8.28 ± 0.92 ^bc^	69.99 ± 2.21 ^a^	0.62 ± 0.01 ^a^	1.03 ± 0.04 ^ab^
*Cistus parviflorus*	Leaves	2.44 ± 0.03 ^b^	5.38 ± 0.87 ^cd^	68.03 ± 1.61 ^ab^	0.61 ± 0.02 ^a^	1.07 ± 0.03 ^ab^
	Roots	2.53 ± 0.01 ^a^	10.90 ± 0.62 ^ab^	71.19 ± 1.34 ^a^	0.65 ± 0.04 ^a^	1.08 ± 0.01 ^ab^
	Twigs	2.52 ± 0.02 ^a^	8.63 ± 1.11 ^ab^	71.46 ± 1.38 ^a^	0.64 ± 0.02 ^a^	1.09 ± 0.03 ^a^

Values are reported as mean ± SD of three parallel measurements. GALAE: Galantamine equivalent; KAE: Kojic acid equivalent; ACAE: Acarbose equivalent. Different letters indicate significant differences between the tested extracts (*p* < 0.05).

**Table 6:** Minimal inhibitory concentrations (MICs) of *C. monspeliensis* and *C. parviflorus* extracts against bacteria isolates.

		MIC (µg/mL)			
*Cistus* Species	Parts	*Escherichia coli* (ATCC 10536)	*Pseudomonas aeruginosa* (ATCC 15442)	*Bacillus subtilis* (PeruMycA 6)	*Salmonella typhi* (PeruMycA 7)
*Cistus monspeliensis*	Leaves	7.87	15.75	31.50	39.68
	Roots	15.75	15.75	62.99	62.99
	Twigs	3.96	7.77	79.37	125.99
*Cistus parviflorus*	Leaves	7.87	9.92	31.50	79.37
	Roots	7.87	15.75	39.68	158
	Twigs	15.75	9.92	158	125.99
Standard	Ciprofloxacin (µg/mL)	31.49	125.99	125.99	79.37

MIC values are reported as geometric means of three independent replicates (n = 3).

**Table 7:** Minimal inhibitory concentrations of *C. monspeliensis* and *C. parviflorus* extracts against yeast isolates.

		MIC (μg/mL)		
*Cistus* Species	Parts	*Candida tropicalis* (YEPGA 6184)	*Candida albicans* (YEPGA 6379)	*Candida parapsilosis* (YEPGA 6551)
*Cistus monspeliensis*	Leaves	125.99	31.50	125.99
	Roots	62.99	158.74	158.74
	Twigs	62.99	79.37	62.99
*Cistus parviflorus*	Leaves	79.37	39.68	31.50
	Roots	39.68	31.50	62.99
	Twigs	15.75	39.68	31.50
Standard	Fluconazole (μg/mL)	2	1	4

**Table 8:** Minimal inhibitory concentrations (MICs) of *C. monspeliensis* and *C. parviflorus* extract against dermatophyte isolates.

		MIC (µg/mL)					
Cistus Species	Extracts	*Trichophyton mentagrophytes* (CCF 4823)	*Trichophyton tonsurans* (CCF 4834)	*Arthroderma quadrifidum*	*Arthroderma insingulare* (CCF 5417)	*Trichophyton mentagrophytes* (CCF 5930)	*Auxarthron ostraviense* DB7
*Cistus monspeliensis*	Leaves	125.99	31.49	79.37	62.99	125.99	125.99
	Roots	>200	62.99	125.99	125.99	158.74	79.37
	Twigs	>200	158.74	>200	>200	>200	79.37
*Cistus parviflorus*	Leaves	62.99	62.99	39.68	31.49	62.99	125.99
	Roots	>200	>200	125.99	79.37	158.74	125.99
	Twigs	>200	125.99	125.99	>200	158.74	158.74
Standard	Griseofulvin (µg/mL)	2.52	0.198	>8	>8	3.174	3.17

**Table 9:** Cytotoxic effect of *C. monspelialis* and *C. parviflorus* extract.

*Cistus* Species	Extracts	RAW	HepG2	S17
	0.5% DMSO	87.7 ± 5.5	99.7 ± 5.7	99.3 ± 7.2
*Cistus monspeliasis*	Leaves	60.1 ± 3.8	48.8 ± 1.2	98.3 ± 8.9
	Roots	70.9 ± 4.7	105 ± 7	109 ± 8
	Twigs	73.5 ± 5.1	97 ± 6.6	105 ± 8
*Cistus parviflorus*	Leaves	58.1 ± 4.8	82.6 ± 4.4	102 ± 9
	Roots	71.4 ± 4.9	60.3 ± 2.3	112 ± 8
	Twigs	73.8 ± 5	82.6 ± 4	107 ± 8

Extracts were tested at 100 µg/mL, and results are expressed as a percentage of cellular viability (%) relative to the control containing 0.5% DMSO. Values represent the mean ± standard error of the mean (SEM).

## Data Availability

No new data were created or analyzed in this study.

## Supplementary Materials

The following supporting information can be downloaded at: https://www.mdpi.com/article/10.3390/pathogens13090795/s1, Table S1: Chemical composition of the leaves of *C. monspeliensis*; Table S2: Chemical composition of the twigs of *C. monspeliensis*; Table S3: Chemical composition of the roots of *C. monspeliensis*; Table S4: Chemical composition of the leaves of *C. parviflorus*; Table S5: Chemical composition of the twigs of *C. parviflorus*; Table S6: Chemical composition of the roots of *C. parviflorus*; Figure S1: *Cistus monspeliensis* leaves TIC positive; Figure S2: *Cistus monspeliensis* leaves TIC negative; Figure S3: *Cistus monspeliensis* twigs TIC positive; Figure S4: *Cistus monspeliensis* twigs TIC negative; Figure S5: *Cistus monspeliensis* root TIC positive; Figure S6: *Cistus monspeliensis* root TIC negative; Figure S7: *Cistus parviflorus* leaves TIC positive; Figure S8: *Cistus parviflorus* leaves TIC negative; Figure S9: *Cistus parviflorus* twigs TIC positive; Figure S10: *Cistus parviflorus* twigs TIC negative; Figure S11: *Cistus parviflorus* root TIC positive; Figure S12: *Cistus parviflorus* root TIC negative.
