# Supplementary material for: Characterising the Metabolomic Diversity and Biological Potentials of Extracts from Different Parts of Two Cistus Species Using UHPLC-MS/MS and In Vitro Techniques
Source: Pathogens. 2024 Sep 13;13(9):795. doi: 10.3390/pathogens13090795 (PMC11435373; doi:10.3390/pathogens13090795)

UHPLC-MS/MS analysis

Analysis of different extracts was carried out on liquid chromatography coupled with mass spectrometry (UHPLC/MS/MS) using a system in which UHPLC (Dionex Ultimate 3000RS) system was equipped with Mass Spectrometer (Q-Exactive Orbitrap, Thermo, USA).

Before the analysis extracts were filtered through 0.22 µm PTFE filter membrane (Labex Ltd, Hungary). In order to get chromatographic separation, 2 µL of each sample was injected into the HPLC system equipped with reverse phase C-18 column (Accucore C18 (100 mm x 2.1, mm i. d., 2.6 µm, Thermo). Column was themostated at 25 °C (± 1 °C). The elution was carried out at a flow rate of 0.2 mL/min using gradient elution. The solvents used were water (A) and methanol (B). Both was acidified with 0.1 % formic acid. Elution was performed using following gradient: isocratic 5 % B (0-3 min), a linear gradient increasing from 5% B to 100% (3-43 min), 100% B (43-61 min), a linear gradient decreasing from 100% B to 5% (61-62 min) and 5 % B (62-70 min). The total run time of analysis was 70 minutes.

The Thermo Q-Exactive Orbitrap mass spectrometer equipped with electrospray ionization source was in positive or negative polarity at the resolving power of 70,000 (full MS, range: m/z 100-1500) and 35000 (ddMS^2). The ESI source parameters include ion spray voltage 4.0 kV in positive and 3.8 kV in negative mode; capillary temperature 320 °C; S-lens RF level 50 V; auxiliary gas: N2 (purity > 95%), heater temperature 300 °C. The data was acquired in full MS-ddMS^2 mode by using Xcalibur 3.1 software. The full MS-ddMS^2 mode provided a full MS with MS/MS spectrum simultaneously in a single LC run. The full MS spectrum provided information about the intact molecular ion (e.g., M^+, $[M + H]^+$, $[M - H]^-$), while the ddMS^2 discovery generates the product ion spectra. The acquired data was processed by using TraceFinder 3.1 software (Thermo Fisher Scientific). All detailed analytical conditions have been also published (Zengin et al., 2018). Two runs were made with each sample, spectra were recorded separately in positive and negative mode. This method helped the identification of compounds in comparison with databases and peak identification was also based on the comparision the chromatographic data with standards, the exact molecular mass/adducts, fragmentation patterns, isotopic distributions and comparision with own tandem mass spectral library (MS/MS). As can be seen from the data in Tables (next chapter) a large number of

components, structural isomers were tentatively identified. All samples were measured in both positive and negative ionization mode, the data recorded in the negative mode were more suitable for identifying the components.

Antibacterial Susceptibility Testing

The Minimum Inhibitory Concentration (MIC) of the *Cistus* extracts was evaluated using the broth dilution method M07-A9 as outlined by the Clinical and Laboratory Standards Institute [31]. To determine MIC, bacterial suspensions were prepared by selecting three to five colonies of the bacterial strains from 24-hour cultures on Tryptic Soy Agar plates (TSA). These colonies were then cultured overnight in Mueller-Hinton Broth (MHB) to achieve a cell density of approximately 1-2 x 10^8 Colony-Forming Units (CFU) per milliliter, which is equivalent to the 0.5 McFarland standard. The bacterial suspensions were subsequently diluted in fresh MHB and added to the MIC dilution series to reach a concentration of 5 x 10^5 CFU per milliliter in each tube, following CLSI M07-A9 guidelines. This was confirmed by plating serial dilutions of the inoculum suspensions on Mueller-Hinton agar (MHA). Control groups consisted of MHB-grown bacterial cultures for viability assessment, as well as uninoculated MHB supplemented with *Cistus* extracts for incubation.

MIC endpoints were determined after 18-20 hours of incubation at 35°C in ambient air, following the method described by Pagiotti et al. [28]. Geometric means and MIC ranges were calculated from three biological replicates to enable comparisons among the activities of *Cistus* spp. extracts.

Antifungal Susceptibility Testing

Susceptibility testing for yeasts and filamentous fungi followed the CLSI M27-A3 and M38-A2 protocols [30-33]. The study utilized Roswell Park Memorial Institute (RPMI, Sigma) 1640 medium, which was sodium bicarbonate-free and enriched with 2% glucose (w/v), L-glutamine, and buffered with 0.165 mol L-1 morpholinepropanesulfonic acid at pH 7.0. In brief, fungal inocula were prepared from 7-day-old cultures on Sabouraud dextrose agar (SDA; Difco) at 25°C and adjusted to optical densities (OD 600) between 0.09 and 0.11 (MacFarland standard). Inoculum sizes ranging from 0.2 to 0.4 x $10^{4–5}$ CFU mL^{-1} were achieved by diluting filamentous fungi and yeast inoculum suspensions to a 1:50 ratio in RPMI 1640. The accuracy of the inoculum sizes was confirmed by plating serial dilutions on SDA. MIC endpoints (µg dry extracts mL^{-1}) were determined after 24 hours (for yeasts) and 72 hours (for dermatophytes) of incubation at 30°C in ambient air [30-33]. Geometric means and MIC ranges were calculated from three biological replicates to allow for comparison of the effectiveness of *Cistus* extracts.

Supplementary Table S1

Chemical composition of the leaves of *C. monspeliensis*

No.	Name	Formula	Rt	$[M + H]^+$	$[M - H]^-$	Fragment 1	Fragment 2	Fragment 3	Fragment 4	Fragment 5
1	Quinic acid	C7H12O6	2,07		191,05557	173,0448	171,0289	111,0437	109,0281	85,0280
2	Malic acid	C4H6O5	2,13		133,01370	115,0022	89,0228	87,0072	72,9916	71,0122
3	Citric acid	C6H8O7	3,04		191,01918	173,0085	129,0182	111,0073	87,0072	85,0280
4[1]	Gallic acid (3,4,5-Trihydroxybenzoic acid)	C7H6O5	4,54		169,01370	125,0230	97,0280	81,0330	79,0172	69,0330
5	Gentisic acid (2,5-Dihydroxybenzoic acid)	C7H6O4	8,98		153,01879	109,0281	108,0202	81,0331		
6	Gallocatechin	C15H14O7	10,17		305,06613	261,0763	219,0659	167,0338	137,0231	125,0231
7	Gentisic acid-O-glucoside	C13H16O9	10,41		315,07161	153,0181	152,0103	109,0281	108,0202	
8	Flavogallonic acid dilactone or isomer	C21H10O13	14,26		469,00432	425,0154	407,0042	379,0101	351,0151	299,9916
9	Uralenneoside	C12H14O8	14,72		285,06105	153,0181	152,0103	109,0281	108,0202	
10	Esculin (Esculetin-6-O-glucoside)	C15H16O9	15,05	341,08726		179,0339	151,0391	133,0287	123,0442	85,0288
11[1]	Catechin	C15H14O6	15,90		289,07121	245,0818	203,0708	151,0389	125,0231	109,0281
12[1]	Epigallocatechin	C15H14O7	15,92		305,06613	261,0769	219,0658	167,0339	137,0232	125,0231
13	Scopolin (Scopoletin-7-O-glucoside)	C16H18O9	16,49	355,10291		193,0496	178,0262	165,0546	137,0597	133,0285
14	Esculetin (6,7-Dihydroxycoumarin)	C9H6O4	16,99	179,03444		151,0391	133,0286	123,0443	117,0342	105,0338
15	Monspelioside (1-(3,5-Dihydroxy-2-methylphenyl)ethanone-5-O-glucoside)	C15H20O8	18,46		327,10800	165,0546	147,0439	113,0230	101,0230	89,0228
16[1]	Epicatechin	C15H14O6	19,10		289,07121	245,0818	203,0710	151,0387	125,0230	109,0281
17	Fraxetin (7,8-Dihydroxy-6-methoxycoumarin)	C10H8O5	19,38	209,04500		194,0208	181,0493	153,0546	149,0231	135,0441
18[1]	p-Coumaric acid	C9H8O3	20,70		163,03952	119,0488				
19	Vicenin-2 (Apigenin-6,8-di-C-glucoside)	C27H30O15	20,86	595,16630		577,1552	457,1132	379,0810	325,0706	295,0602
20[1]	Scopoletin (7-Hydroxy-6-methoxycoumarin)	C10H8O4	20,97	193,05009		178,0260	165,0546	149,0600	137,0598	133,0285
21[1]	Taxifolin (Dihydroquercetin)	C15H12O7	21,80		303,05048	285,0408	217,0500	175,0391	151,0025	125,0231
22	Ellagic acid-4-O-glucoside	C20H16O13	22,25		463,05127	300,9994	299,9914	257,0079		

23[1]	Sinapic acid	C11H12O5	22,27	225,07630		207,0653	192,0419	175,0390	147,0440	119,0494
24	Dimethoxy-hydroxycoumarin	C11H10O5	22,51	223,06065		208,0365	207,0288	190,0260	179,0337	162,0310
25	Myricetin-3-O-glucoside (Isomyricitrin)	C21H20O13	23,22		479,08257	317,0304	316,0226	287,0202	271,0251	178,9979
26	Scoparone (6,7-Dimethoxycoumarin)	C11H10O4	23,38	207,06574		192,0414	191,0340	179,0701	151,0754	136,0520
27	Myricetin-O-pentoside isomer 1	C20H18O12	23,50		449,07201	317,0309	316,0226	287,0198	271,0250	214,0268
28	Myricetin-O-pentoside isomer 2	C20H18O12	24,30		449,07201	317,0305	316,0226	287,0201	271,0251	214,0265
29	Dihydrokaempferol (3,4',5,7-Tetrahydroxyflavanone)	C15H12O6	24,31		287,05557	269,0464	259,0613	243,0660	177,0549	125,0231
30[1]	Myricitrin (Myricetin-3-O-rhamnoside)	C21H20O12	24,40		463,08765	317,0305	316,0226	287,0201	271,0251	178,9977
31	Quercetin-O-pentosylhexoside	C26H28O16	24,42		595,12992	301,0355	300,0277	271,0251	255,0299	151,0024
32	Myricetin-O-pentoside isomer 3	C20H18O12	24,49		449,07201	317,0304	316,0226	287,0201	271,0252	214,0268
33[1]	Hyperoside (Quercetin-3-O-galactoside)	C21H20O12	25,05		463,08765	301,0355	300,0278	271,0251	255,0298	151,0024
34	Trimethoxycoumarin	C12H12O5	25,14	237,07630		222,0523	207,0289	193,0499	191,0342	176,0465
35	Ellagic acid-O-pentoside	C19H14O12	25,23		433,04071	300,9991	299,9913	283,9959	257,0086	
36[1]	Rutin (Quercetin-3-O-rutinoside)	C27H30O16	25,30		609,14557	301,0355	300,0276	271,0247	255,0301	151,0026
37	Eschweilenol A or isomer	C20H16O12	25,74		425,01449	300,9992	299,9914			
38	Ellagic acid	C14H6O8	26,17		300,99845	283,9967	257,0089	229,0137	201,0189	185,0238
39	Kaempferol-7-O-glucoside	C21H20O11	26,49		447,09274	285,0408	284,0329	255,0299	227,0346	151,0021
40[1]	Myricetin (3,3',4',5,5',7-Hexahydroxyflavone)	C15H10O8	26,79		317,02974	271,0241	178,9978	151,0025	137,0232	109,0282
41[1]	Quercitrin (Quercetin-3-O-rhamnoside)	C21H20O11	26,81		447,09274	301,0355	300,0278	271,0251	255,0298	151,0025
42	Pinobanksin (3,5,7-Trihydroxyflavanone)	C15H12O5	29,00		271,06065	253,0506	225,0549	197,0602	151,0026	125,0232
43	Quercetin-O-coumaroylhexoside	C30H26O14	29,15		609,12444	463,0891	301,0356	300,0278	271,0252	255,0301
44[1]	Quercetin (3,3',4',5,7-Pentahydroxyflavone)	C15H10O7	29,25		301,03483	273,0412	178,9977	151,0025	121,0281	107,0125
45	Trihydroxy-trimethoxy(iso)flavone-O-hexoside	C24H26O13	29,68	523,14517		361,0917	346,0678	345,0599	331,0450	
46[1]	Luteolin (3',4',5,7-Tetrahydroxyflavone)	C15H10O6	30,25		285,03991	217,0502	199,0392	175,0391	151,0025	133,0282
47	Tiliroside (6''-O-trans-p-Coumaroylastragalin)	C30H26O13	30,54		593,12952	447,0951	285,0410	284,0330	255,0299	227,0346
48	Quercetin-3-O-methyl ether	C16H12O7	30,57		315,05048	300,0278	271,0251	255,0299	243,0298	227,0334
49	Dimethoxy-tetrahydroxy(iso)flavone	C17H14O8	30,88		345,06105	330,0384	315,0151	287,0198	271,0252	259,0249
50[1]	Kaempferol (3,4',5,7-Tetrahydroxyflavone)	C15H10O6	31,47		285,03991	257,0461	229,0489	185,0607	151,0026	107,0123

51	Isorhamnetin-7-O-rhamnoside	C22H22O11	31,64		461,10839	315,0515	314,0437	300,0277	299,0204	271,0255
52[1]	Isorhamnetin (3'-Methoxy-3,4',5,7-tetrahydroxyflavone)	C16H12O7	31,92		315,05048	300,0279	283,0252	227,0337	164,0103	151,0025
53[1]	Apigenin (4',5,7-Trihydroxyflavone)	C15H10O5	32,00		269,04500	227,0346	225,0553	151,0025	149,0233	117,0332
54	Chrysoeriol (3'-Methoxy-4',5,7-trihydroxyflavone)	C16H12O6	32,27		299,05556	284,0329	256,0378	255,0293	227,0345	151,0026
55	Isokaempferide (3-Methoxy-4',5,7-trihydroxyflavone)	C16H12O6	32,59	301,07122		286,0471	285,0392	258,0523	229,0484	213,0539
56	Dimethoxy-trihydroxy(iso)flavone	C17H14O7	32,83		329,06613	314,0436	299,0200	285,0408	271,0251	243,0297
57	Rhamnetin (7-Methoxy-3,3',4',5-tetrahydroxyflavone)	C16H12O7	33,38		315,05048	300,0278	193,0138	165,0183	121,0281	97,0281
58	Trihydroxy-trimethoxy(iso)flavone isomer 1	C18H16O8	33,45		359,07670	344,0539	329,0305	314,0071	301,0358	286,0124
59	Pinocembrin (5,7-Dihydroxyflavanone)	C15H12O4	33,69		255,06573	227,0696	213,0553	151,0026	145,0647	107,0125
60	Luteolin-7-O-methyl ether (7-Methoxy-3',4',5-trihydroxyflavone)	C16H12O6	34,13		299,05556	284,0329	256,0376	227,0342	151,0025	133,0280
61	Trihydroxy-trimethoxy(iso)flavone isomer 2	C18H16O8	34,43		359,07670	344,0541	329,0307	314,0071	301,0356	285,0408
62	Dihydroxy-trimethoxy(iso)flavone isomer 1	C18H16O7	34,66	345,09743		330,0733	329,0652	315,0497	299,0554	287,0550
63	Dihydroxy(iso)flavone	C15H10O4	34,91		253,05009	225,0548	209,0602	151,0023	143,0489	107,0124
64	Methoxy-trihydroxy(iso)flavone isomer 1	C16H12O6	35,53	301,07122		286,0467	258,0520	227,0703	179,0342	167,0342
65	Acacetin (5,7-Dihydroxy-4'-methoxyflavone)	C16H12O5	35,56		283,06065	268,0380	240,0424	239,0348	211,0390	151,0024
66	Methoxy-trihydroxy(iso)flavone isomer 2	C16H12O6	35,73	301,07122		286,0471	258,0523	230,0575	153,0182	
67[1]	Genkwanin (4',5-Dihydroxy-7-methoxyflavone)	C16H12O5	35,87	285,07630		270,0520	242,0572	167,0337	119,0492	
68	Kumatakenin (4',5-Dihydroxy-3,7-dimethoxyflavone)	C17H14O6	35,95		313,07122	298,0485	297,0388	283,0250	270,0537	255,0298
69	Dihydroxy-trimethoxy(iso)flavone isomer 2	C18H16O7	36,22	345,09743		330,0733	329,0652	315,0497	301,0710	287,0549
70	Ermanin (5,7-Dihydroxy-3,4'-dimethoxyflavone)	C17H14O6	36,42		313,07122	298,0487	283,0252	269,0454	255,0299	227,0343
71	Dihydroxy-trimethoxy(iso)flavone isomer 3	C18H16O7	36,46	345,09743		330,0732	329,0654	315,0495	301,0707	287,0546
72	Myricetin-3,3',4',7-tetramethyl ether (5,5'-Dihydroxy-3,3',4',7-tetramethoxyflavone)	C19H18O8	36,68		373,09235	358,0699	343,0461	328,0230	315,0513	285,0043
73	Vitexilactone or isomer	C22H34O5	37,45	379,24845		361,2371	319,2267	301,2159	283,2054	255,2100
74	Hydroxy-tetramethoxy(iso)flavone	C19H18O7	37,99	359,11308		344,0888	343,0807	329,0654	315,0844	301,0705
75	Hydroxy-methoxy(iso)flavone	C16H12O4	38,33	269,08138		254,0575	226,0625	167,0340		
76	Apigenin-4',7-dimethyl ether (4',7-Dimethoxy-5-hydroxyflavone)	C17H14O5	38,96	299,09195		284,0680	256,0729	167,0335	133,0646	
77	Kaempferol-3,4',7-trimethyl ether (5-Hydroxy-3,4',7-trimethoxyflavone)	C18H16O6	39,77	329,10252		314,0782	313,0702	299,0549	285,0758	271,0599
78	18-Hydroxy-cis-clerodan-3-ene-15-oic acid or 15-Hydroxy-cis-clerodan-3-ene-18-oic acid	C20H32O3	40,74		319,22732	275,2382	259,2062	237,1850		
79	Cistadiol (15,18-Dihydroxy-cis-clerodan-3-ene)	C20H36O2	41,62	309,27936		291,2680	273,2553	221,1516	163,1481	95,0859

80	18-Acetoxy-cis-clerodan-3-ene-15-oic acid or 15-Acetoxy-cis-clerodan-3-ene-18-oic acid	C22H36O4	42,98		363,25353	321,2439	303,2334	59,0123		
81	8-Hydroxylabdan-15-oic acid	C20H36O3	43,34		323,25862	305,2507	279,2700	263,2378	247,2059	
82	Pheophytin A	C55H74N4O5	65,85	871,57375		593,2756	533,2546	505,2190	460,2252	433,2413

Supplementary Table S2

Chemical composition of the twigs of *C. monspeliensis*

No.	Name	Formula	Rt	$[M + H]^+$	$[M - H]^-$	Fragment 1	Fragment 2	Fragment 3	Fragment 4
1	Quinic acid	C7H12O6	2,06		191,05557	173,0448	171,0289	111,0437	109,0281
2	Malic acid	C4H6O5	2,23		133,01370	115,0022	89,0228	87,0072	72,9915
3	Citric acid	C6H8O7	3,05		191,01918	173,0079	129,0180	111,0073	87,0072
4[1]	Gallic acid (3,4,5-Trihydroxybenzoic acid)	C7H6O5	4,53		169,01370	125,0230	97,0282	81,0331	79,0172
5	Gentisic acid (2,5-Dihydroxybenzoic acid)	C7H6O4	9,04		153,01879	109,0280	108,0202	81,0329	
6	Gallocatechin	C15H14O7	10,17		305,06613	261,0771	219,0656	167,0337	137,0231
7	Gentisic acid-O-glucoside	C13H16O9	10,44		315,07161	153,0180	152,0101	109,0280	108,0201
8	Procyanidin B isomer 1	C30H26O12	14,13		577,13460	451,1034	425,0875	407,0771	289,0720
9	Flavogallonic acid dilactone or isomer	C21H10O13	14,28		469,00432	425,0152	407,0032	379,0089	351,0118
10	Uralenneoside	C12H14O8	14,73		285,06105	153,0180	152,0102	109,0280	108,0202
11	Procyanidin B isomer 2	C30H26O12	14,74		577,13460	451,1028	425,0883	407,0773	289,0721
12	Esculin (Esculetin-6-O-glucoside)	C15H16O9	15,08	341,08726		179,0338	151,0393	133,0284	123,0440
13[1]	Catechin	C15H14O6	15,91		289,07121	245,0817	203,0708	151,0387	125,0230
14[1]	Epigallocatechin	C15H14O7	15,93		305,06613	261,0769	219,0654	167,0338	137,0231
15	Procyanidin B isomer 3	C30H26O12	15,96		577,13460	451,1054	425,0893	407,0780	289,0717
16	Magnolioside (Isoscopoletin-6-O-glucoside)	C16H18O9	16,11	355,10291		193,0495	178,0261	165,0552	137,0597
17	Scopolin (Scopoletin-7-O-glucoside)	C16H18O9	16,53	355,10291		193,0496	178,0258	165,0547	137,0598
18	Esculetin (6,7-Dihydroxycoumarin)	C9H6O4	17,01	179,03444		151,0389	133,0283	123,0442	117,0342
19	Procyanidin B isomer 4	C30H26O12	17,31		577,13460	451,1018	425,0882	407,0771	289,0718
20	Fraxetin-O-glucoside	C16H18O10	18,00		369,08218	207,0291	206,0214	192,0055	190,9977

21	Monspelioside (1-(3,5-Dihydroxy-2-methylphenyl)ethanone-5-O-glucoside)	C15H20O8	18,48		327,10800	165,0544	147,0439	113,0228	101,0230
22	Naringenin-6,8-di-C-glucoside	C27H32O15	18,75		595,16630	505,1369	475,1250	415,1019	385,0928
23[1]	Epicatechin	C15H14O6	19,12		289,07121	245,0816	203,0705	151,0388	125,0230
24	Fraxetin (7,8-Dihydroxy-6-methoxycoumarin)	C10H8O5	19,42	209,04500		194,0206	181,0488	153,0543	149,0233
25	Isoscopoletin (6-Hydroxy-7-methoxycoumarin)	C10H8O4	20,32	193,05009		178,0261	165,0546	149,0594	137,0598
26[1]	p-Coumaric acid	C9H8O3	20,72		163,03952	119,0487			
27	Vicenin-2 (Apigenin-6,8-di-C-glucoside)	C27H30O15	20,89	595,16630		577,1539	457,1122	379,0814	325,0704
28[1]	Scopoletin (7-Hydroxy-6-methoxycoumarin)	C10H8O4	21,02	193,05009		178,0260	165,0546	149,0597	137,0598
29[1]	Taxifolin (Dihydroquercetin)	C15H12O7	21,82		303,05048	285,0405	217,0494	175,0388	151,0026
30	Ellagic acid-4-O-glucoside	C20H16O13	22,28		463,05127	300,9990	299,9908	257,0079	
31	Dimethoxy-hydroxycoumarin	C11H10O5	22,57	223,06065		208,0365	207,0289	190,0261	179,0337
32	Myricetin-3-O-glucoside (Isomyricitrin)	C21H20O13	23,30		479,08257	317,0302	316,0223	287,0200	271,0248
33	Myricetin-O-pentoside isomer 1	C20H18O12	23,54		449,07201	317,0329	316,0224	287,0195	271,0247
34	Myricetin-O-pentoside isomer 2	C20H18O12	24,36		449,07201	317,0301	316,0223	287,0197	271,0245
35	Dihydrokaempferol (3,4',5,7-Tetrahydroxyflavanone)	C15H12O6	24,37		287,05557	269,0457	259,0611	243,0666	177,0549
36[1]	Myricitrin (Myricetin-3-O-rhamnoside)	C21H20O12	24,44		463,08765	317,0300	316,0223	287,0197	271,0250
37	Myricetin-O-pentoside isomer 3	C20H18O12	24,51		449,07201	317,0302	316,0224	287,0199	271,0252
38[1]	Hyperoside (Quercetin-3-O-galactoside)	C21H20O12	25,09		463,08765	301,0356	300,0276	271,0246	255,0300
39[1]	Isoquercitrin (Quercetin-3-O-glucoside)	C21H20O12	25,25		463,08765	301,0355	300,0276	271,0250	255,0290
40	Ellagic acid-O-pentoside	C19H14O12	25,27		433,04071	300,9992	299,9912	283,9959	257,0086
41	Ellagic acid	C14H6O8	26,19		300,99845	283,9970	257,0091	229,0136	201,0188
42	Kaempferol-7-O-glucoside	C21H20O11	26,52		447,09274	285,0411	284,0323	255,0298	227,0337
43[1]	Myricetin (3,3',4',5,5',7-Hexahydroxyflavone)	C15H10O8	26,82		317,02974	271,0238	178,9978	151,0024	137,0235
44[1]	Quercitrin (Quercetin-3-O-rhamnoside)	C21H20O11	26,84		447,09274	301,0354	300,0276	271,0250	255,0296
45	3-O-Methylellagic acid-4'-O-rhamnoside	C21H18O12	27,89		461,07201	315,0147	299,9911	298,9813	270,9897
46	Pinobanksin (3,5,7-Trihydroxyflavanone)	C15H12O5	29,03		271,06065	253,0504	225,0552	197,0603	151,0024
47	Quercetin-O-coumaroylhexoside	C30H26O14	29,18		609,12444	463,0886	301,0354	300,0275	271,0249
48[1]	Naringenin (4',5,7-Trihydroxyflavanone)	C15H12O5	29,20		271,06065	177,0183	165,0178	151,0023	119,0487

49[1]	Quercetin (3,3',4',5,7-Pentahydroxyflavone)	C15H10O7	29,31		301,03483	273,0408	178,9976	151,0023	121,0280
50	Trihydroxy-trimethoxy(iso)flavone-O-hexoside	C24H26O13	29,72	523,14517		361,0915	346,0677	345,0603	331,0444
51	3,4'-Di-O-methylellagic acid	C16H10O8	30,23		329,02975	314,0072	298,9837	270,9887	
52[1]	Luteolin (3',4',5,7-Tetrahydroxyflavone)	C15H10O6	30,30		285,03991	217,0500	199,0400	175,0394	151,0023
53	3,3'-Di-O-methylellagic acid	C16H10O8	30,48		329,02975	314,0070	298,9830	270,9890	
54	Tiliroside (6''-O-trans-p-Coumaroylastragalin)	C30H26O13	30,57		593,12952	447,0935	285,0406	284,0328	255,0296
55	Quercetin-3-O-methyl ether	C16H12O7	30,61		315,05048	300,0275	271,0249	255,0295	243,0297
56	Dimethoxy-tetrahydroxy(iso)flavone	C17H14O8	30,92		345,06105	330,0383	315,0147	287,0199	271,0252
57[1]	Kaempferol (3,4',5,7-Tetrahydroxyflavone)	C15H10O6	31,53		285,03991	257,0461	229,0498	185,0595	151,0025
58[1]	Isorhamnetin (3'-Methoxy-3,4',5,7-tetrahydroxyflavone)	C16H12O7	31,99		315,05048	300,0276	283,0252	227,0341	164,0101
59[1]	Apigenin (4',5,7-Trihydroxyflavone)	C15H10O5	32,03		269,04500	227,0341	225,0549	151,0023	149,0230
60	Chrysoeriol (3'-Methoxy-4',5,7-trihydroxyflavone)	C16H12O6	32,30		299,05556	284,0326	256,0376	255,0291	227,0343
61	Isokaempferide (3-Methoxy-4',5,7-trihydroxyflavone)	C16H12O6	32,63	301,07122		286,0472	285,0393	258,0519	229,0482
62	Dimethoxy-trihydroxy(iso)flavone	C17H14O7	32,88		329,06613	314,0432	299,0196	285,0407	271,0248
63	Rhamnetin (7-Methoxy-3,3',4',5-tetrahydroxyflavone)	C16H12O7	33,41		315,05048	300,0278	193,0135	165,0181	121,0280
64	Trihydroxy-trimethoxy(iso)flavone isomer 1	C18H16O8	33,47		359,07670	344,0538	329,0302	314,0067	301,0354
65	Pinocembrin (5,7-Dihydroxyflavanone)	C15H12O4	33,71		255,06573	227,0707	213,0550	151,0024	145,0645
66	Luteolin-7-O-methyl ether (7-Methoxy-3',4',5-trihydroxyflavone)	C16H12O6	34,16		299,05556	284,0333	256,0374	227,0342	151,0024
67	Trihydroxy-trimethoxy(iso)flavone isomer 2	C18H16O8	34,47		359,07670	344,0536	329,0302	314,0059	301,0352
68	Dihydroxy-trimethoxy(iso)flavone isomer 1	C18H16O7	34,70	345,09743		330,0732	329,0652	315,0498	299,0552
69	Dihydroxy(iso)flavone	C15H10O4	34,92		253,05009	225,0544	209,0598	151,0026	143,0489
70	Methoxy-trihydroxy(iso)flavone isomer 1	C16H12O6	35,57	301,07122		286,0468	258,0528	227,0706	179,0344
71	Acacetin (5,7-Dihydroxy-4'-methoxyflavone)	C16H12O5	35,59		283,06065	268,0377	240,0421	239,0336	211,0396
72	Methoxy-trihydroxy(iso)flavone isomer 2	C16H12O6	35,80	301,07122		286,0472	258,0519	230,0566	153,0177
73[1]	Genkwanin (4',5-Dihydroxy-7-methoxyflavone)	C16H12O5	35,93	285,07630		270,0521	242,0574	167,0335	119,0493
74	Kumatakenin (4',5-Dihydroxy-3,7-dimethoxyflavone)	C17H14O6	35,97		313,07122	298,0483	297,0386	283,0252	270,0527
75	Dihydroxy-trimethoxy(iso)flavone isomer 2	C18H16O7	36,27	345,09743		330,0732	329,0660	315,0497	301,0724
76	Ermanin (5,7-Dihydroxy-3,4'-dimethoxyflavone)	C17H14O6	36,46		313,07122	298,0482	283,0249	269,0449	255,0297
77	Dihydroxy-trimethoxy(iso)flavone isomer 3	C18H16O7	36,52	345,09743		330,0732	329,0656	315,0500	301,0701

78	Myricetin-3,3',4',7-tetramethyl ether (5,5'-Dihydroxy-3,3',4',7-tetramethoxyflavone)	C19H18O8	36,72		373,09235	358,0696	343,0458	328,0207	315,0521
79	Vitexilactone or isomer	C22H34O5	37,50	379,24845		361,2374	319,2270	301,2162	283,2057
80	Hydroxy-tetramethoxy(iso)flavone	C19H18O7	38,05	359,11308		344,0889	343,0811	329,0656	315,0868
81	Hydroxy-methoxy(iso)flavone	C16H12O4	38,38	269,08138		254,0565	226,0624	167,0342	
82	Apigenin-4',7-dimethyl ether (4',7-Dimethoxy-5-hydroxyflavone)	C17H14O5	39,02	299,09195		284,0676	256,0729	167,0343	133,0651
83	Kaempferol-3,4',7-trimethyl ether (5-Hydroxy-3,4',7-trimethoxyflavone)	C18H16O6	39,81	329,10252		314,0783	313,0703	299,0550	285,0754
84	18-Hydroxy-cis-clerodan-3-ene-15-oic acid or 15-Hydroxy-cis-clerodan-3-ene-18-oic acid	C20H32O3	40,76		319,22732	275,2385	259,2065	237,1856	
85	Cistadiol (15,18-Dihydroxy-cis-clerodan-3-ene)	C20H36O2	41,65	309,27936		291,2679	273,2551	221,1510	163,1480
86	18-Acetoxy-cis-clerodan-3-ene-15-oic acid or 15-Acetoxy-cis-clerodan-3-ene-18-oic acid	C22H36O4	43,01		363,25353	321,2422	303,2330	59,0122	
87	8-Hydroxylabdan-15-oic acid	C20H36O3	43,34		323,25862	305,2489	279,2697	263,2376	247,2065
88	Pheophytin A	C55H74N4O5	65,87	871,57375		593,2764	533,2551	505,2212	460,2260

Supplementary Table S3

Chemical composition of the roots of *C. monspeliensis*

No.	Name	Formula	Rt	$[M + H]^+$	$[M - H]^-$	Fragment 1	Fragment 2	Fragment 3	Fragment 4	Fragment 5
1	Quinic acid	C7H12O6	2,06		191,05557	173,0442	171,0293	111,0437	109,0280	85,0279
2	Malic acid	C4H6O5	2,27		133,01370	115,0023	89,0229	87,0071	72,9916	71,0123
3	Citric acid	C6H8O7	3,08		191,01918	173,0078	129,0179	111,0074	87,0072	85,0280
4[1]	Gallic acid (3,4,5-Trihydroxybenzoic acid)	C7H6O5	4,54		169,01370	125,0230	97,0281	81,0331	79,0172	69,0329
5	Gentisic acid (2,5-Dihydroxybenzoic acid)	C7H6O4	9,05		153,01879	109,0280	108,0202	81,0331		
6	Gallocatechin	C15H14O7	10,19		305,06613	261,0772	219,0658	167,0339	137,0231	125,0230
7	Gentisic acid-O-glucoside	C13H16O9	10,41		315,07161	153,0180	152,0102	109,0280	108,0202	
8	Procyanidin B isomer 1	C30H26O12	14,14		577,13460	451,1037	425,0884	407,0774	289,0721	125,0230
9	Uralenneoside	C12H14O8	14,73		285,06105	153,0181	152,0102	109,0280	108,0202	
10	Procyanidin B isomer 2	C30H26O12	14,74		577,13460	451,1030	425,0877	407,0772	289,0719	125,0230
11	Unidentified hydroxybenzoic acid derivative 1	C21H24O11	15,17		451,12404	313,0932	287,0569	161,0232	137,0232	125,0230
12[1]	Catechin	C15H14O6	15,91		289,07121	245,0816	203,0706	151,0388	125,0231	109,0280
13[1]	Epigallocatechin	C15H14O7	15,92		305,06613	261,0767	219,0656	167,0338	137,0231	125,0230
14	Procyanidin B isomer 3	C30H26O12	15,99		577,13460	451,1050	425,0874	407,0775	289,0721	125,0231
15	Magnolioside (Isoscopoletin-6-O-glucoside)	C16H18O9	16,11	355,10291		193,0497	178,0257	165,0552	137,0597	133,0285
16	Scopolin (Scopoletin-7-O-glucoside)	C16H18O9	16,53	355,10291		193,0496	178,0262	165,0546	137,0596	133,0283
17	Procyanidin B isomer 4	C30H26O12	17,31		577,13460	451,1001	425,0880	407,0767	289,0722	125,0230
18	Monspelioside (1-(3,5-Dihydroxy-2-methylphenyl)ethanone-5-O-glucoside)	C15H20O8	18,45		327,10800	165,0544	147,0440	113,0230	101,0231	89,0230
19	Unidentified hydroxybenzoic acid derivative 2	C22H26O11	18,54		465,13969	301,0720	286,0482	166,0260	137,0232	125,0231
20	Naringenin-6,8-di-C-glucoside	C27H32O15	18,73		595,16630	505,1374	475,1250	415,1031	385,0950	355,0818
21[1]	Epicatechin	C15H14O6	19,11		289,07121	245,0817	203,0707	151,0388	125,0230	109,0281
22	Isoscopoletin (6-Hydroxy-7-methoxycoumarin)	C10H8O4	20,31	193,05009		178,0258	165,0545	149,0594	137,0600	133,0285
23[1]	p-Coumaric acid	C9H8O3	20,72		163,03952	119,0488				
24	Vicenin-2 (Apigenin-6,8-di-C-glucoside)	C27H30O15	20,90	595,16630		577,1524	457,1128	379,0813	325,0708	295,0604

25[1]	Scopoletin (7-Hydroxy-6-methoxycoumarin)	C10H8O4	21,00	193,05009		178,0261	165,0546	149,0599	137,0599	133,0285
26	Dimethoxy-hydroxycoumarin	C11H10O5	22,57	223,06065		208,0369	207,0288	190,0262	179,0338	162,0312
27	Myricetin-3-O-glucoside (Isomyricitrin)	C21H20O13	23,30		479,08257	317,0302	316,0224	287,0205	271,0252	178,9972
28[1]	Myricitrin (Myricetin-3-O-rhamnoside)	C21H20O12	24,43		463,08765	317,0299	316,0225	287,0191	271,0250	178,9976
29[1]	Hyperoside (Quercetin-3-O-galactoside)	C21H20O12	25,09		463,08765	301,0355	300,0291	271,0242	255,0296	151,0018
30[1]	Isoquercitrin (Quercetin-3-O-glucoside)	C21H20O12	25,28		463,08765	301,0360	300,0277	271,0254	255,0287	151,0023
31	Ellagic acid	C14H6O8	26,21		300,99845	283,9965	257,0088	229,0133	201,0190	185,0235
32[1]	Quercitrin (Quercetin-3-O-rhamnoside)	C21H20O11	26,82		447,09274	301,0353	300,0277	271,0253	255,0302	151,0026
33	3-O-Methylellagic acid-4'-O-rhamnoside	C21H18O12	27,86		461,07201	315,0151	299,9915	298,9811	270,9894	
34[1]	Quercetin (3,3',4',5,7-Pentahydroxyflavone)	C15H10O7	29,33		301,03483	273,0410	178,9976	151,0024	121,0281	107,0125
35[1]	Luteolin (3',4',5,7-Tetrahydroxyflavone)	C15H10O6	30,28		285,03991	217,0520	199,0395	175,0397	151,0029	133,0281
36	3,3'-Di-O-methylellagic acid	C16H10O8	30,50		329,02975	314,0071	298,9835	270,9886		
37	Quercetin-3-O-methyl ether	C16H12O7	30,60		315,05048	300,0276	271,0245	255,0289	243,0294	227,0334
38[1]	Isorhamnetin (3'-Methoxy-3,4',5,7-tetrahydroxyflavone)	C16H12O7	31,99		315,05048	300,0276	283,0252	227,0337	164,0105	151,0024
39[1]	Apigenin (4',5,7-Trihydroxyflavone)	C15H10O5	32,01		269,04500	227,0341	225,0537	151,0026	149,0227	117,0330
40	Chrysoeriol (3'-Methoxy-4',5,7-trihydroxyflavone)	C16H12O6	32,32		299,05556	284,0328	256,0387	255,0290	227,0341	151,0024
41	Isokaempferide (3-Methoxy-4',5,7-trihydroxyflavone)	C16H12O6	32,59	301,07122		286,0470	285,0390	258,0521	229,0483	213,0536
42	Dimethoxy-trihydroxy(iso)flavone	C17H14O7	32,87		329,06613	314,0437	299,0199	285,0406	271,0252	243,0297
43	Rhamnetin (7-Methoxy-3,3',4',5-tetrahydroxyflavone)	C16H12O7	33,43		315,05048	300,0271	193,0136	165,0182	121,0281	97,0282
44	Trihydroxy-trimethoxy(iso)flavone isomer 1	C18H16O8	33,47		359,07670	344,0538	329,0304	314,0066	301,0360	286,0121
45	Malyngic acid (9,12,13-Trihydroxy-10E,15Z-octadecadienoic acid)	C18H32O5	33,63		327,21715	309,2069	291,1967	229,1440	211,1332	171,1015
46	Pinocembrin (5,7-Dihydroxyflavanone)	C15H12O4	33,70		255,06573	227,0695	213,0549	151,0024	145,0647	107,0123
47	Luteolin-7-O-methyl ether (7-Methoxy-3',4',5-trihydroxyflavone)	C16H12O6	34,17		299,05556	284,0328	256,0374	227,0341	151,0022	133,0281
48	Trihydroxy-trimethoxy(iso)flavone isomer 2	C18H16O8	34,47		359,07670	344,0543	329,0300	314,0068	301,0363	285,0415
49	Pinellic acid (9,12,13-Trihydroxy-10E-octadecenoic acid)	C18H34O5	34,83		329,23280	311,2226	293,2128	229,1440	211,1334	99,0801
50	Ermanin (5,7-Dihydroxy-3,4'-dimethoxyflavone)	C17H14O6	36,46		313,07122	298,0484	283,0251	269,0458	255,0297	227,0341
51	Myricetin-3,3',4',7-tetramethyl ether (5,5'-Dihydroxy-3,3',4',7-tetramethoxyflavone)	C19H18O8	36,72		373,09235	358,0695	343,0459	328,0220	315,0512	285,0043
52	Vitexilactone or isomer	C22H34O5	37,50	379,24845		361,2369	319,2268	301,2161	283,2054	255,2097

53	Emodin	C15H10O5	39,54		269,04500	241,0504	225,0552	197,0594			
54	18-Hydroxy-cis-clerodan-3-ene-15-oic acid or 15-Hydroxy-cis-clerodan-3-ene-18-oic acid	C20H32O3	40,75		319,22732	275,2384	259,2069	237,1857			
55	18-Hydroxy-cis-clerodan-3-ene-15-oic acid or 15-Hydroxy-cis-clerodan-3-ene-18-oic acid	C20H32O3	41,33		319,22732	275,2383	259,2072	259,2072			
56	18-Acetoxy-cis-clerodan-3-ene-15-oic acid or 15-Acetoxy-cis-clerodan-3-ene-18-oic acid	C22H36O4	43,00		363,25353	321,2439	303,2332	59,0123			
57	8-Hydroxylabdan-15-oic acid	C20H36O3	43,35		323,25862	305,2504	279,2695	263,2372	247,2055		

Supplementary Table S4

Chemical composition of the leaves of *C. parviflorus*

No.	Name	Formula	Rt	$[M + H]^+$	$[M - H]^-$	Fragment 1	Fragment 2	Fragment 3	Fragment 4	Fragment 5
1	Quinic acid	C7H12O6	2,06		191,05557	173,0444	171,0287	127,0388	111,0436	85,0279
2	Citric acid	C6H8O7	2,99		191,01918	173,0076	129,0180	111,0073	87,0072	85,0279
3	Arbutin	C12H16O7	3,12		271,08178	161,0447	151,0385	108,0202	101,0231	85,0280
4[1]	Gallic acid (3,4,5-Trihydroxybenzoic acid)	C7H6O5	4,56		169,01370	125,0230	97,0281	81,0330	79,0173	69,0329
5	Protocatechuic acid (3,4-Dihydroxybenzoic acid)	C7H6O4	9,01		153,01879	110,0322	109,0280	108,0203	91,0174	81,0331
6	Gallocatechin	C15H14O7	10,20		305,06613	261,0762	179,0341	167,0338	137,0231	125,0230
7	Punicalagin isomer	C48H28O30	11,96		1083,05872	781,0549	600,9895	575,0088	300,9988	270,9888
8	Flavogallonic acid dilactone or isomer	C21H10O13	14,24		469,00432	425,0154	407,0047	379,0097	351,0147	299,9916
9	Punicalagin	C48H28O30	14,64		1083,05872	781,0565	600,9907	575,0087	300,9990	270,9883
10	Esculin (Esculetin-6-O-glucoside)	C15H16O9	15,08	341,08726		179,0338	151,0386	133,0285	123,0450	85,0289
11	Unidentified hydroxybenzoic acid derivative 1	C21H24O11	15,16		451,12404	313,0937	287,0564	161,0232	137,0231	125,0230
12[1]	Catechin	C15H14O6	15,91		289,07121	245,0816	203,0706	151,0387	125,0231	109,0280
13[1]	Epigallocatechin	C15H14O7	15,92		305,06613	261,0768	179,0342	167,0339	137,0232	125,0231
14	Caffeic acid-O-glucoside	C15H18O9	17,00		341,08726	179,0341	135,0438			
15[1]	Caffeic acid	C9H8O4	17,54		179,03444	135,0440	107,0490			
16	Unidentified hydroxybenzoic acid derivative 2	C21H24O10	18,71		435,12912	313,0929	289,0719	271,0608	137,0231	125,0229
17	Naringenin-6,8-di-C-glucoside	C27H32O15	18,74		595,16630	505,1333	475,1219	415,1034	385,0932	355,0828
18[1]	Epigallocatechin-3-O-gallate (Teatannin II)	C22H18O11	18,90		457,07709	331,0459	305,0664	193,0139	169,0131	125,0230
19[1]	Epicatechin	C15H14O6	19,11		289,07121	245,0817	203,0707	151,0388	125,0231	109,0280
20	Isoscopoletin (6-Hydroxy-7-methoxycoumarin)	C10H8O4	20,31	193,05009		178,0259	165,0554	149,0593	137,0599	133,0285
21[1]	p-Coumaric acid	C9H8O3	20,72		163,03952	119,0487				
22	Vicenin-2 (Apigenin-6,8-di-C-glucoside)	C27H30O15	20,89	595,16630		577,1534	511,1252	457,1127	325,0705	295,0599
23[1]	Scopoletin (7-Hydroxy-6-methoxycoumarin)	C10H8O4	21,02	193,05009		178,0259	165,0552	149,0595	137,0597	133,0284
24	Methyl flavogallonate	C22H12O13	21,13		483,01997	450,9944	432,9822	407,0043	367,0097	125,0228
25	Ellagic acid-4-O-glucoside	C20H16O13	22,28		463,05127	300,9991	299,9916	283,9962	257,0092	
26	Quercetin-O-dirhamnosylhexoside	C33H40O20	22,99		755,20347	301,0359	300,0277	271,0251	255,0301	151,0024
27	Myricetin-3-O-glucoside (Isomyricitrin)	C21H20O13	23,26		479,08257	317,0301	316,0225	287,0200	271,0250	178,9978

28	Quercetin-O-galloylhexoside	C28H24O16	23,89		615,09862	463,0887	301,0340	300,0275	271,0244	125,0225
29	Kaempferol-O-dirhamnosylhexoside	C33H40O19	24,25		739,20856	285,0406	284,0327	255,0297	227,0344	151,0021
30	Myricetin-3-O-pentoside	C20H18O12	24,33		449,07201	317,0299	316,0225	287,0205	271,0253	178,9974
31[1]	Myricitrin (Myricetin-3-O-rhamnoside)	C21H20O12	24,45		463,08765	317,0304	316,0222	287,0207	271,0255	178,9976
32	Kaempferol-3-O-neohesperidoside	C27H30O15	24,59		593,15065	429,0826	327,0529	285,0406	284,0327	255,0297
33	Myricetin-O-malonylhexoside	C24H22O16	24,88		565,08297	521,0932	316,0229	287,0213	271,0255	
34[1]	Hyperoside (Quercetin-3-O-galactoside)	C21H20O12	25,07		463,08765	301,0356	300,0277	271,0250	255,0297	151,0024
35	Ellagic acid-O-pentoside	C19H14O12	25,23		433,04071	300,9991	299,9912	298,9806	283,9965	257,0095
36[1]	Isoquercitrin (Quercetin-3-O-glucoside)	C21H20O12	25,25		463,08765	301,0354	300,0276	271,0250	255,0298	151,0024
37[1]	Rutin (Quercetin-3-O-rutinoside)	C27H30O16	25,31		609,14557	301,0356	300,0277	271,0250	255,0297	151,0023
38	Avicularin (Quercetin-3-O-arabinofuranoside)	C20H18O11	25,96		433,07709	301,0353	300,0275	271,0250	255,0298	151,0024
39	Ellagic acid	C14H6O8	26,16		300,99845	283,9965	257,0085	245,0089	229,0139	201,0187
40	Kaempferol-7-O-glucoside	C21H20O11	26,50		447,09274	285,0405	284,0329	255,0297	227,0344	151,0025
41	Quercetin-O-malonylhexoside	C24H22O15	26,52		549,08805	505,0994	301,0354	300,0277	271,0252	255,0299
42	Guaijaverin (Quercetin-3-O-arabinopyranoside)	C20H18O11	26,56		433,07709	301,0354	300,0279	271,0255	255,0298	151,0028
43	Dimethoxy-trihydroxyflavone-O-hexoside	C23H24O12	26,58		491,11896	476,0962	461,0721	328,0589	313,0355	299,0200
44[1]	Astragalin (Kaempferol-3-O-glucoside)	C21H20O11	26,99		447,09274	327,0515	285,0406	284,0327	255,0297	227,0343
45	Kaempferol-3-O-rutinoside (Nicotiflorin)	C27H30O15	27,11		593,15065	327,0524	285,0407	284,0328	255,0298	227,0344
46	Isorhamnetin-3-O-rutinoside (Narcissin)	C28H32O16	27,47		623,16122	315,0515	314,0443	300,0272	299,0202	271,0254
47	Kaempferol-O-malonylhexoside	C24H22O14	28,32		533,09314	489,1039	285,0407	284,0328	255,0294	227,0347
48	Pinobanksin (3,5,7-Trihydroxyflavanone)	C15H12O5	29,04		271,06065	253,0504	225,0544	215,0708	197,0605	151,0024
49[1]	Naringenin (4',5,7-Trihydroxyflavanone)	C15H12O5	29,21		271,06065	177,0187	165,0184	151,0024	119,0488	107,0122
50[1]	Quercetin (3,3',4',5,7-Pentahydroxyflavone)	C15H10O7	29,31		301,03483	273,0407	229,0508	178,9977	151,0024	107,0124
51	Kaempferol-3-O-[rhamnosyl-(1→2)-(6''-O-trans-p-coumaroyl)]glucoside	C36H36O17	29,66		739,18743	593,1525	285,0406	284,0328	255,0297	227,0344
52	Tiliroside (6''-O-trans-p-Coumaroylastragalin)	C30H26O13	30,57		593,12952	447,0928	285,0406	284,0328	255,0297	227,0344
53	Quercetin-3-O-methyl ether	C16H12O7	30,62		315,05048	300,0277	271,0250	255,0298	243,0295	227,0335
54	3''-O-trans-p-Coumaroylastragalin	C30H26O13	30,82		593,12952	447,0917	285,0406	284,0328	255,0297	227,0345
55	Axillarin (3,6-Dimethoxy-3',4',5,7-tetrahydroxyflavone)	C17H14O8	30,92		345,06104	330,0382	315,0148	287,0199	271,0254	259,0248
56[1]	Kaempferol (3,4',5,7-Tetrahydroxyflavone)	C15H10O6	31,52		285,03991	257,0441	229,0512	213,0548	185,0600	151,0022
57[1]	Isorhamnetin (3'-Methoxy-3,4',5,7-tetrahydroxyflavone)	C16H12O7	31,99		315,05048	300,0277	283,0252	271,0255	164,0103	151,0024
58[1]	Apigenin (4',5,7-Trihydroxyflavone)	C15H10O5	32,03		269,04500	227,0337	225,0544	151,0022	149,0231	117,0329

59[1]	Chrysoeriol (3'-Methoxy-4',5,7-trihydroxyflavone)	C16H12O6	32,27		299,05556	284,0329	256,0375	227,0329	151,0022	
60	Isokaempferide (3-Methoxy-4',5,7-trihydroxyflavone)	C16H12O6	32,65	301,07122		286,0472	285,0394	258,0521	213,0550	121,0285
61	3,8-Dimethoxy-4',5,7-trihydroxyflavone	C17H14O7	32,87		329,06613	314,0435	299,0199	285,0406	271,0251	243,0296
62	Rhamnetin (7-Methoxy-3,3',4',5-tetrahydroxyflavone)	C16H12O7	33,43		315,05048	300,0276	193,0136	165,0181	151,0021	121,0281
63	Jaceidin (4',5,7-Trihydroxy-3,3',6-trimethoxyflavone)	C18H16O8	33,48	361,09235		346,0680	345,0603	331,0445	315,0490	275,0547
64	Pinocembrin (5,7-Dihydroxyflavanone)	C15H12O4	33,70		255,06573	227,0711	213,0553	151,0024	145,0647	107,0124
65	3,6-Dimethoxy-4',5,7-trihydroxyflavone	C17H14O7	34,41		329,06613	314,0434	299,0198	285,0390	271,0250	243,0294
66	4',5,7-Trihydroxy-3,3',8-trimethoxyflavone (Gossypetin-3,3',8-trimethyl ether)	C18H16O8	34,46	361,09235		346,0681	345,0605	331,0446	315,0492	303,0498
67	Kaempferol-3-O-(3,6-di-p-coumaroylglucoside)	C39H32O15	34,64		739,16630	593,1315	447,0958	285,0407	284,0329	255,0298
68	Dihydroxy-trimethoxy(iso)flavone isomer 1	C18H16O7	34,72	345,09743		330,0731	329,0653	315,0497	299,0552	287,0548
69	Dihydroxy-trimethoxy(iso)flavone isomer 2	C18H16O7	36,28	345,09743		330,0732	329,0654	315,0497	301,0713	287,0552
70	Dihydroxy-tetramethoxy(iso)flavone	C19H18O8	36,33	375,10800		360,0839	359,0766	345,0607	329,0651	317,0654
71	5,7-Dihydroxy-3,4',8-trimethoxyflavone (Herbacetin-3,4',8-trimethyl ether)	C18H16O7	36,51	345,09743		330,0731	329,0652	315,0496	301,0698	287,0552
72	5,7-Dihydroxy-3,3',4',8-tetramethoxyflavone (Gossypetin-3,3',4',8-tetramethyl ether)	C19H18O8	36,72	375,10800		360,0837	359,0758	345,0604	330,0383	314,0423
73	Flindulatin (5-Hydroxy-3,4',7,8-tetramethoxyflavone)	C19H18O7	38,05	359,11308		344,0889	343,0814	329,0656	315,0864	301,0708
74	Kaempferol-3,4',7-trimethyl ether (5-Hydroxy-3,4',7-trimethoxyflavone)	C18H16O6	39,82	329,10252		314,0781	313,0702	299,0544	285,0760	271,0597
75	Pheophytin A	C55H74N4O5	65,85	871,57375		593,2758	533,2546	505,2172	460,2253	433,2400

Supplementary Table S5

Chemical composition of the twigs of *C. parviflorus*

No.	Name	Formula	Rt	$[M + H]^+$	$[M - H]^-$	Fragment 1	Fragment 2	Fragment 3	Fragment 4	Fragment 5
1	Quinic acid	C7H12O6	2,05		191,05557	173,0441	171,0285	127,0387	111,0437	85,0279
2	Citric acid	C6H8O7	3,02		191,01918	173,0081	129,0180	111,0073	87,0072	85,0279
3	Arbutin	C12H16O7	3,16		271,08178	161,0441	151,0388	108,0202	101,0229	85,0278
4	Prodelphinidin B isomer 1	C30H26O14	4,08		609,12444	441,0822	423,0726	305,0668	177,0183	125,0230
1[5]	Gallic acid (3,4,5-Trihydroxybenzoic acid)	C7H6O5	4,52		169,01370	125,0230	97,0280	81,0330	79,0172	69,0329
6	Prodelphinidin B isomer 2	C30H26O14	5,61		609,12444	441,0843	423,0727	305,0667	177,0183	125,0230
7	Protocatechuic acid (3,4-Dihydroxybenzoic acid)	C7H6O4	9,00		153,01879	110,0311	109,0280	108,0202	91,0174	81,0330
8	Prodelphinidin B isomer 3	C30H26O14	9,31		609,12444	441,0824	423,0725	305,0668	177,0183	125,0229
9	Gallocatechin	C15H14O7	10,16		305,06613	261,0767	179,0341	167,0338	137,0232	125,0230
10	Prodelphinidin B isomer 4	C30H26O14	11,62		609,12444	441,0831	423,0723	305,0669	177,0181	125,0230
11	Procyanidin B isomer 1	C30H26O12	14,16		577,13460	451,1033	425,0880	407,0773	289,0720	125,0230
12	Flavogallonic acid dilactone or isomer	C21H10O13	14,28		469,00432	425,0151	407,0045	379,0089	351,0161	299,9915
13	Prodelphinidin B isomer 5	C30H26O14	14,72		609,12444	441,0842	423,0726	305,0668	177,0183	125,0231
14	Procyanidin B isomer 2	C30H26O12	14,75		577,13460	451,1038	425,0875	407,0773	289,0721	125,0231
15	Esculin (Esculetin-6-O-glucoside)	C15H16O9	15,08	341,08726		179,0338	151,0391	133,0285	123,0441	85,0289
16	Unidentified hydroxybenzoic acid derivative 1	C21H24O11	15,17		451,12404	313,0930	287,0566	161,0231	137,0231	125,0230
17[1]	Catechin	C15H14O6	15,91		289,07121	245,0815	203,0706	151,0387	125,0230	109,0280
18[1]	Epigallocatechin	C15H14O7	15,93		305,06613	261,0768	179,0340	167,0336	137,0231	125,0230
19	Magnolioside (Isoscopoletin-6-O-glucoside)	C16H18O9	16,11	355,10291		193,0496	178,0262	165,0553	137,0594	133,0286
20	Scopolin (Scopoletin-7-O-glucoside)	C16H18O9	16,54	355,10291		193,0496	178,0259	165,0544	137,0597	133,0286
21	Caffeic acid-O-glucoside	C15H18O9	16,97		341,08726	179,0340	135,0438			
22[1]	Caffeic acid	C9H8O4	17,54		179,03444	135,0439	107,0491			
23	Unidentified hydroxybenzoic acid derivative 2	C21H24O10	18,74		435,12912	313,0932	289,0724	271,0611	137,0231	125,0230
24	Naringenin-6,8-di-C-glucoside	C27H32O15	18,76		595,16630	505,1338	475,1234	415,1073	385,0929	355,0828
25[1]	Epigallocatechin-3-O-gallate (Teatannin II)	C22H18O11	18,90		457,07709	331,0476	305,0671	193,0135	169,0130	125,0230
26[1]	Epicatechin	C15H14O6	19,13		289,07121	245,0815	203,0706	151,0388	125,0229	109,0280
27	Isoscopoletin (6-Hydroxy-7-methoxycoumarin)	C10H8O4	20,32	193,05009		178,0258	165,0552	149,0592	137,0596	133,0284

28[1]	p-Coumaric acid	C9H8O3	20,73		163,03952	119,0487					
29	Vicenin-2 (Apigenin-6,8-di-C-glucoside)	C27H30O15	20,91	595,16630		577,1538	511,1228	457,1119	325,0703	295,0599	
30[1]	Scopoletin (7-Hydroxy-6-methoxycoumarin)	C10H8O4	21,02	193,05009		178,0259	165,0544	149,0595	137,0597	133,0285	
31	Methyl flavogallonate	C22H12O13	21,14		483,01997	450,9961	432,9883	407,0040	367,0091	125,0226	
32	Ellagic acid-4-O-glucoside	C20H16O13	22,29		463,05127	300,9994	299,9918	283,9960	257,0088		
33	Quercetin-O-dirhamnosylhexoside	C33H40O20	23,02		755,20347	301,0357	300,0277	271,0248	255,0304	151,0024	
34	Myricetin-3-O-glucoside (Isomyricitrin)	C21H20O13	23,29		479,08257	317,0297	316,0224	287,0197	271,0248	178,9983	
35	Kaempferol-O-dirhamnosylhexoside	C33H40O19	24,29		739,20856	285,0406	284,0327	255,0294	227,0346	151,0016	
36	Myricetin-3-O-pentoside	C20H18O12	24,36		449,07201	317,0296	316,0223	287,0192	271,0254	178,9980	
37	3-O-Methylellagic acid-4'-O-glucoside	C21H18O13	24,39		477,06692	315,0150	314,0067	299,9908	298,9837	270,9894	
38[1]	Myricitrin (Myricetin-3-O-rhamnoside)	C21H20O12	24,45		463,08765	317,0295	316,0224	287,0213	271,0256	178,9985	
39	Kaempferol-3-O-neohesperidoside	C27H30O15	24,62		593,15065	429,0821	327,0526	285,0405	284,0328	255,0297	
40[1]	Hyperoside (Quercetin-3-O-galactoside)	C21H20O12	25,10		463,08765	301,0353	300,0277	271,0250	255,0299	151,0022	
41	Ellagic acid-O-pentoside	C19H14O12	25,27		433,04071	300,9990	299,9912	298,9832	283,9967	257,0087	
42[1]	Isoquercitrin (Quercetin-3-O-glucoside)	C21H20O12	25,29		463,08765	301,0357	300,0276	271,0248	255,0290	151,0023	
43[1]	Rutin (Quercetin-3-O-rutinoside)	C27H30O16	25,34		609,14557	301,0361	300,0267	271,0251	255,0308	151,0023	
44	Eschweilenol C (Ellagic acid-4-O-rhamnoside)	C20H16O12	25,78		447,05636	300,9992	299,9913				
45	Avicularin (Quercetin-3-O-arabinofuranoside)	C20H18O11	25,99		433,07709	301,0356	300,0268	271,0240	255,0295	151,0024	
46	Ellagic acid	C14H6O8	26,23		300,99845	283,9966	257,0092	245,0082	229,0132	201,0189	
47	Kaempferol-7-O-glucoside	C21H20O11	26,53		447,09274	285,0409	284,0328	255,0302	227,0342	151,0024	
48	Dimethoxy-trihydroxyflavone-O-hexoside	C23H24O12	26,61		491,11896	476,0968	461,0720	328,0595	313,0353	299,0205	
49[1]	Astragalin (Kaempferol-3-O-glucoside)	C21H20O11	27,02		447,09274	327,0523	285,0406	284,0327	255,0297	227,0343	
50	Kaempferol-3-O-rutinoside (Nicotiflorin)	C27H30O15	27,14		593,15065	327,0521	285,0406	284,0328	255,0300	227,0343	
51	Ducheside A (3-O-Methylellagic acid-4'-O-xyloside)	C20H16O12	27,25		447,05636	315,0146	314,0078	299,9916	298,9840	270,9909	
52	3-O-Methylellagic acid-4'-O-rhamnoside	C21H18O12	27,86		461,07201	315,0149	299,9912				
53	3-O-Methylellagic acid	C15H8O8	28,42		315,01410	299,9918	244,0008				
54	Pinobanksin (3,5,7-Trihydroxyflavanone)	C15H12O5	29,04		271,06065	253,0502	225,0541	215,0704	197,0602	151,0023	
55[1]	Naringenin (4',5,7-Trihydroxyflavanone)	C15H12O5	29,23		271,06065	177,0186	165,0184	151,0024	119,0488	107,0124	
56[1]	Quercetin (3,3',4',5,7-Pentahydroxyflavone)	C15H10O7	29,39		301,03483	273,0405	229,0507	178,9971	151,0027	107,0125	
57	Kaempferol-3-O-[rhamnosyl-(1→2)-(6''-O-trans-p-coumaroyl)]glucoside	C36H36O17	29,67		739,18743	593,1481	285,0406	284,0326	255,0296	227,0344	
58	Di-O-methylellagic acid	C16H10O8	30,25		329,02975	314,0071	312,9991	298,9839	270,9882		

59	Tiliroside (6"-O-trans-p-Coumaroylastragalin)	C30H26O13	30,58		593,12952	447,0922	285,0407	284,0328	255,0296	227,0344
60	Quercetin-3-O-methyl ether	C16H12O7	30,62		315,05048	300,0276	271,0250	255,0295	243,0296	227,0334
61	3"-O-trans-p-Coumaroylastragalin	C30H26O13	30,85		593,12952	447,0943	285,0406	284,0328	255,0297	227,0344
62	Axillarin (3,6-Dimethoxy-3',4',5,7-tetrahydroxyflavone)	C17H14O8	30,92		345,06104	330,0382	315,0144	287,0199	271,0252	259,0245
63[1]	Kaempferol (3,4',5,7-Tetrahydroxyflavone)	C15H10O6	31,55		285,03991	257,0440	229,0512	213,0546	185,0599	151,0022
64[1]	Isorhamnetin (3'-Methoxy-3,4',5,7-tetrahydroxyflavone)	C16H12O7	32,05		315,05048	300,0270	283,0250	271,0247	164,0101	151,0021
65[1]	Apigenin (4',5,7-Trihydroxyflavone)	C15H10O5	32,06		269,04500	227,0334	225,0542	151,0022	149,0225	117,0331
66[1]	Chrysoeriol (3'-Methoxy-4',5,7-trihydroxyflavone)	C16H12O6	32,29		299,05556	284,0326	256,0383	227,0326	151,0021	
67	Isokaempferide (3-Methoxy-4',5,7-trihydroxyflavone)	C16H12O6	32,64	301,07122		286,0464	285,0385	258,0519	213,0549	121,0284
68	3,8-Dimethoxy-4',5,7-trihydroxyflavone	C17H14O7	32,89		329,06613	314,0434	299,0200	285,0406	271,0249	243,0296
69	Rhamnetin (7-Methoxy-3,3',4',5-tetrahydroxyflavone)	C16H12O7	33,46		315,05048	300,0285	193,0133	165,0178	151,0021	121,0280
70	Pinocembrin (5,7-Dihydroxyflavanone)	C15H12O4	33,72		255,06573	227,0710	213,0548	151,0024	145,0648	107,0123
71	4',5,7-Trihydroxy-3,3',8-trimethoxyflavone (Gossypetin-3,3',8-trimethyl ether)	C18H16O8	34,46	361,09235		346,0683	345,0605	331,0450	315,0509	303,0496
72	3,6-Dimethoxy-4',5,7-trihydroxyflavone	C17H14O7	34,48		329,06613	314,0440	299,0199	285,0392	271,0256	243,0295
73	Kaempferol-3-O-(3,6-di-p-coumaroylglucoside)	C39H32O15	34,54		739,16630	593,1303	447,0934	285,0406	284,0328	255,0297
74	Dihydroxy-trimethoxy(iso)flavone isomer 2	C18H16O7	36,30	345,09743		330,0732	329,0645	315,0497	301,0715	287,0555
75	Dihydroxy-tetramethoxy(iso)flavone	C19H18O8	36,34	375,10800		360,0838	359,0766	345,0606	329,0659	317,0664
76	5,7-Dihydroxy-3,4',8-trimethoxyflavone (Herbacetin-3,4',8-trimethyl ether)	C18H16O7	36,53	345,09743		330,0732	329,0653	315,0493	301,0720	287,0547
77	5,7-Dihydroxy-3,3',4',8-tetramethoxyflavone (Gossypetin-3,3',4',8-tetramethyl ether)	C19H18O8	36,72	375,10800		360,0837	359,0755	345,0606	330,0380	314,0409
78	Flindulatin (5-Hydroxy-3,4',7,8-tetramethoxyflavone)	C19H18O7	38,06	359,11308		344,0888	343,0817	329,0650	315,0860	301,0708
79	Kaempferol-3,4',7-trimethyl ether (5-Hydroxy-3,4',7-trimethoxyflavone)	C18H16O6	39,84	329,10252		314,0786	313,0707	299,0547	285,0756	271,0597

Supplementary Table S6

Chemical composition of the roots of *C. parviflorus*

No.	Name	Formula	Rt	$[M + H]^+$	$[M - H]^-$	Fragment 1	Fragment 2	Fragment 3	Fragment 4	Fragment 5
1	Quinic acid	C7H12O6	2,06		191,05557	173,0446	171,0293	127,0386	111,0440	85,0279
2	Citric acid	C6H8O7	3,00		191,01918	173,0084	129,0181	111,0072	87,0071	85,0279
3	Arbutin	C12H16O7	3,16		271,08178	161,0445	151,0379	108,0202	101,0230	85,0278
4	Prodelphinidin B isomer 1	C30H26O14	4,11		609,12444	441,0828	423,0723	305,0667	177,0183	125,0230
5[1]	Gallic acid (3,4,5-Trihydroxybenzoic acid)	C7H6O5	4,56		169,01370	125,0230	97,0281	81,0331	79,0171	69,0329
6	Prodelphinidin B isomer 2	C30H26O14	5,59		609,12444	441,0831	423,0724	305,0668	177,0183	125,0230
7	Protocatechuic acid (3,4-Dihydroxybenzoic acid)	C7H6O4	9,01		153,01879	110,0309	109,0280	108,0203	91,0174	81,0329
8	Prodelphinidin B isomer 3	C30H26O14	9,26		609,12444	441,0850	423,0736	305,0671	177,0185	125,0231
9	Gallocatechin	C15H14O7	10,18		305,06613	261,0764	179,0340	167,0337	137,0231	125,0229
10	Prodelphinidin B isomer 4	C30H26O14	11,56		609,12444	441,0830	423,0728	305,0671	177,0183	125,0231
11	Procyanidin B isomer 1	C30H26O12	14,13		577,13460	451,1044	425,0882	407,0773	289,0722	125,0231
12	Prodelphinidin B isomer 5	C30H26O14	14,70		609,12444	441,0830	423,0723	305,0667	177,0185	125,0230
13	Procyanidin B isomer 2	C30H26O12	14,73		577,13460	451,1029	425,0884	407,0772	289,0721	125,0230
14	Esculin (Esculetin-6-O-glucoside)	C15H16O9	15,07	341,08726		179,0337	151,0390	133,0285	123,0441	85,0288
15	Unidentified hydroxybenzoic acid derivative 1	C21H24O11	15,17		451,12404	313,0934	287,0563	161,0232	137,0232	125,0230
16[1]	Catechin	C15H14O6	15,89		289,07121	245,0816	203,0705	151,0388	125,0229	109,0280
17[1]	Epigallocatechin	C15H14O7	15,91		305,06613	261,0776	179,0341	167,0338	137,0232	125,0230
18	Magnolioside (Isoscopoletin-6-O-glucoside)	C16H18O9	16,11	355,10291		193,0496	178,0258	165,0555	137,0606	133,0283
19	Scopolin (Scopoletin-7-O-glucoside)	C16H18O9	16,53	355,10291		193,0496	178,0248	165,0547	137,0603	133,0286
20[1]	Caffeic acid	C9H8O4	17,53		179,03444	135,0439	107,0488			
21	Unidentified hydroxybenzoic acid derivative 2	C21H24O10	18,71		435,12912	313,0924	289,0723	271,0613	137,0231	125,0230
22[1]	Epigallocatechin-3-O-gallate (Teatannin II)	C22H18O11	18,89		457,07709	331,0473	305,0666	193,0136	169,0131	125,0230
23[1]	Epicatechin	C15H14O6	19,10		289,07121	245,0816	203,0707	151,0388	125,0230	109,0280
24	Isoscopoletin (6-Hydroxy-7-methoxycoumarin)	C10H8O4	20,30	193,05009		178,0260	165,0553	149,0590	137,0596	133,0285
25[1]	p-Coumaric acid	C9H8O3	20,71		163,03952	119,0488				

26[1]	Scopoletin (7-Hydroxy-6-methoxycoumarin)	C10H8O4	21,00	193,05009		178,0260	165,0545	149,0597	137,0597	133,0285
27	Myricetin-3-O-glucoside (Isomyricitrin)	C21H20O13	23,32		479,08257	317,0301	316,0223	287,0197	271,0249	178,9977
28[1]	Myricitrin (Myricetin-3-O-rhamnoside)	C21H20O12	24,44		463,08765	317,0322	316,0221	287,0204	271,0232	178,9973
29[1]	Hyperoside (Quercetin-3-O-galactoside)	C21H20O12	25,09		463,08765	301,0358	300,0287	271,0250	255,0294	151,0024
30	Ellagic acid-O-pentoside	C19H14O12	25,25		433,04071	300,9990	299,9915	298,9807	283,9967	257,0096
31[1]	Isoquercitrin (Quercetin-3-O-glucoside)	C21H20O12	25,28		463,08765	301,0365	300,0287	271,0247	255,0286	151,0019
32	Ellagic acid	C14H6O8	26,23		300,99845	283,9968	257,0103	245,0081	229,0136	201,0185
33	Dimethoxy-trihydroxyflavone-O-hexoside	C23H24O12	26,57		491,11896	476,0958	461,0692	328,0582	313,0339	299,0208
34[1]	Astragalin (Kaempferol-3-O-glucoside)	C21H20O11	26,99		447,09274	327,0512	285,0401	284,0328	255,0297	227,0342
35	3-O-Methylellagic acid-4'-O-rhamnoside	C21H18O12	27,84		461,07201	315,0159	299,9912			
36[1]	Naringenin (4',5,7-Trihydroxyflavanone)	C15H12O5	29,20		271,06065	177,0184	165,0182	151,0022	119,0486	107,0124
37[1]	Quercetin (3,3',4',5,7-Pentahydroxyflavone)	C15H10O7	29,36		301,03483	273,0403	229,0505	178,9977	151,0019	107,0124
38	Tiliroside (6''-O-trans-p-Coumaroylastragalin)	C30H26O13	30,56		593,12952	447,0939	285,0407	284,0328	255,0297	227,0345
39	3''-O-trans-p-Coumaroylastragalin	C30H26O13	30,82		593,12952	447,0899	285,0406	284,0328	255,0295	227,0345
40	3,8-Dimethoxy-4',5,7-trihydroxyflavone	C17H14O7	32,88		329,06613	314,0436	299,0191	285,0417	271,0248	243,0296
41	Pinocembrin (5,7-Dihydroxyflavanone)	C15H12O4	33,71		255,06573	227,0710	213,0552	151,0021	145,0653	107,0123
42	3,6-Dimethoxy-4',5,7-trihydroxyflavone	C17H14O7	34,47		329,06613	314,0439	299,0196	285,0393	271,0252	243,0297
43	5,7-Dihydroxy-3,4',8-trimethoxyflavone (Herbacetin-3,4',8-trimethyl ether)	C18H16O7	36,53	345,09743		330,0732	329,0659	315,0506	301,0694	287,0544
44	5,7-Dihydroxy-3,3',4',8-tetramethoxyflavone (Gossypetin-3,3',4',8-tetramethyl ether)	C19H18O8	36,72	375,10800		360,0832	359,0757	345,0601	330,0382	314,0423
45	Flindulatin (5-Hydroxy-3,4',7,8-tetramethoxyflavone)	C19H18O7	38,06	359,11308		344,0884	343,0812	329,0653	315,0864	301,0705

Figure S1. Cistus monspeliensis leaves TIC positive

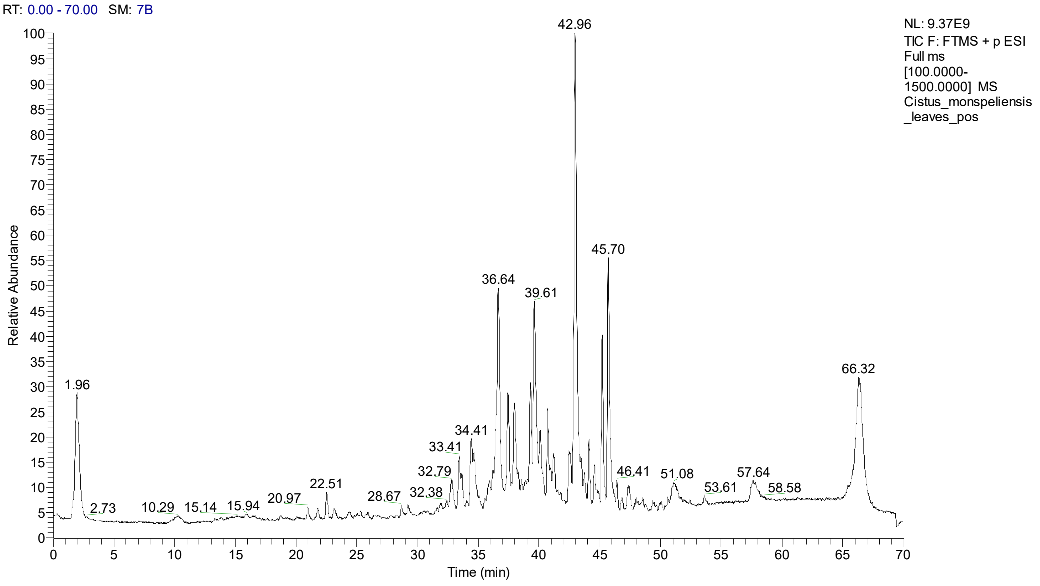

Figure S2. Cistus monspeliensis leaves TIC negative

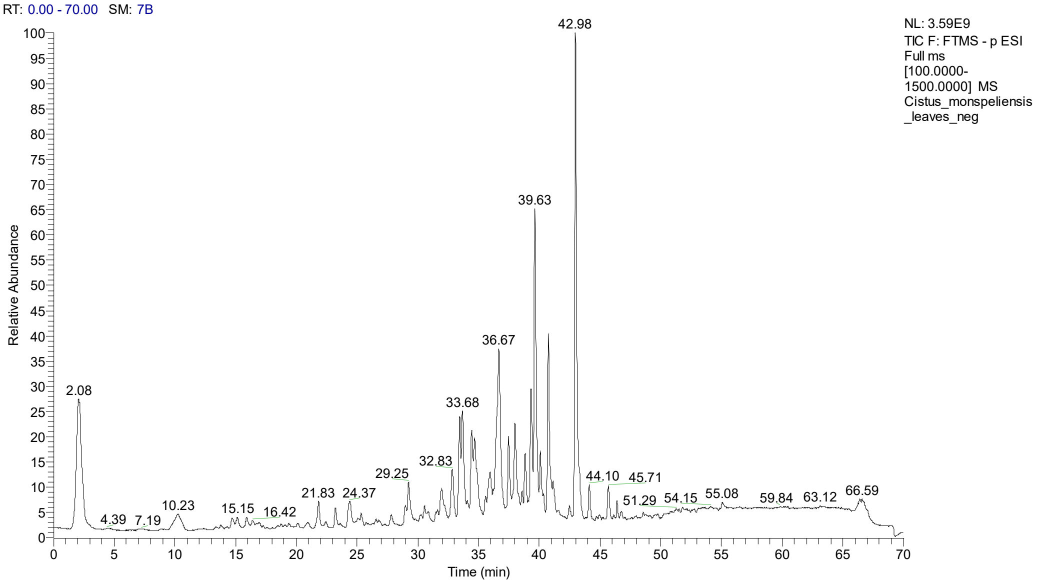

Figure S3. Cistus monspeliensis twigs TIC positive

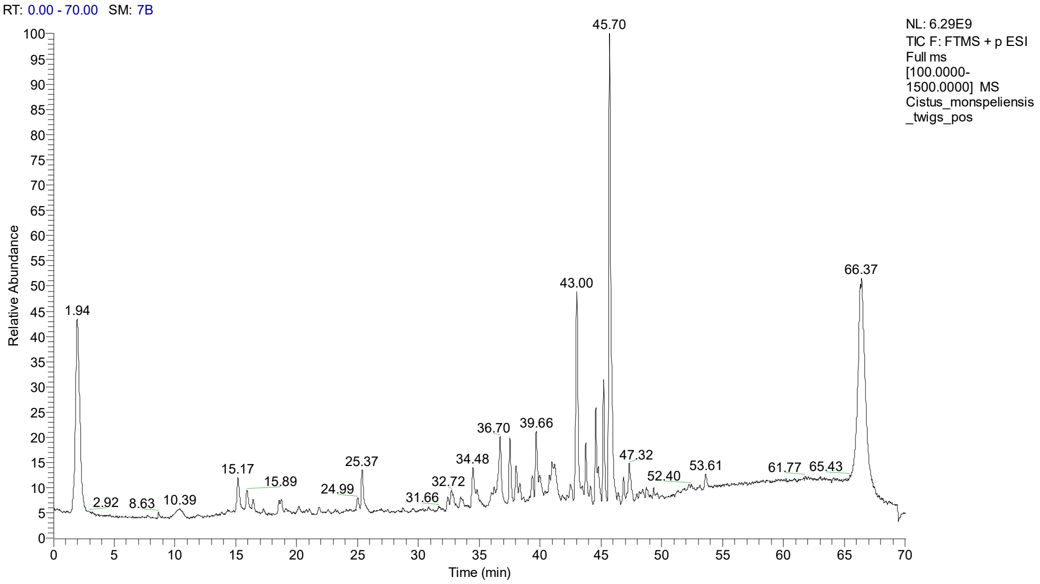

Figure S4. Cistus monspeliensis twigs TIC negative

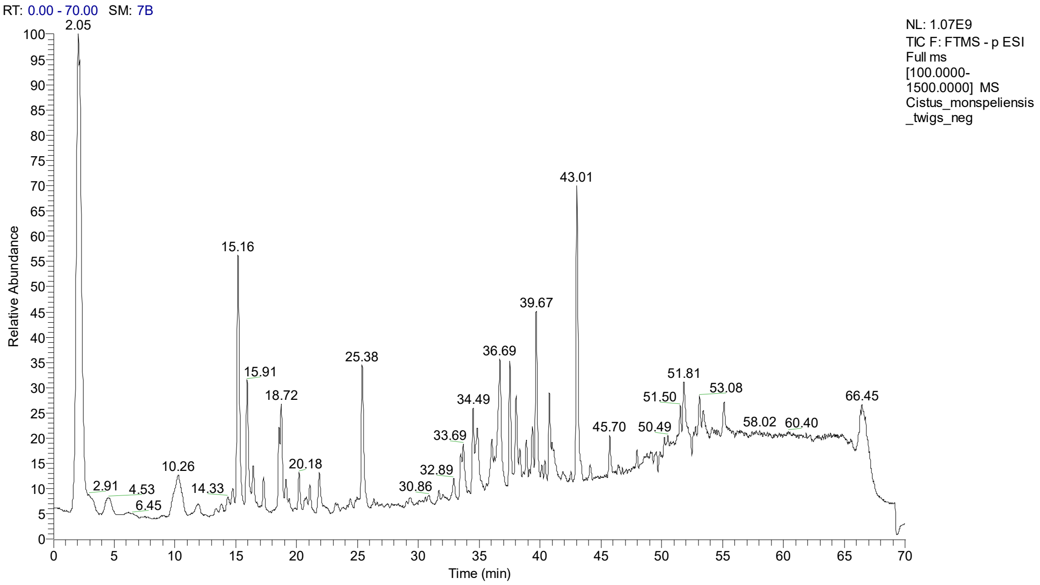

Figure S5. Cistus monspeliensis root TIC positive

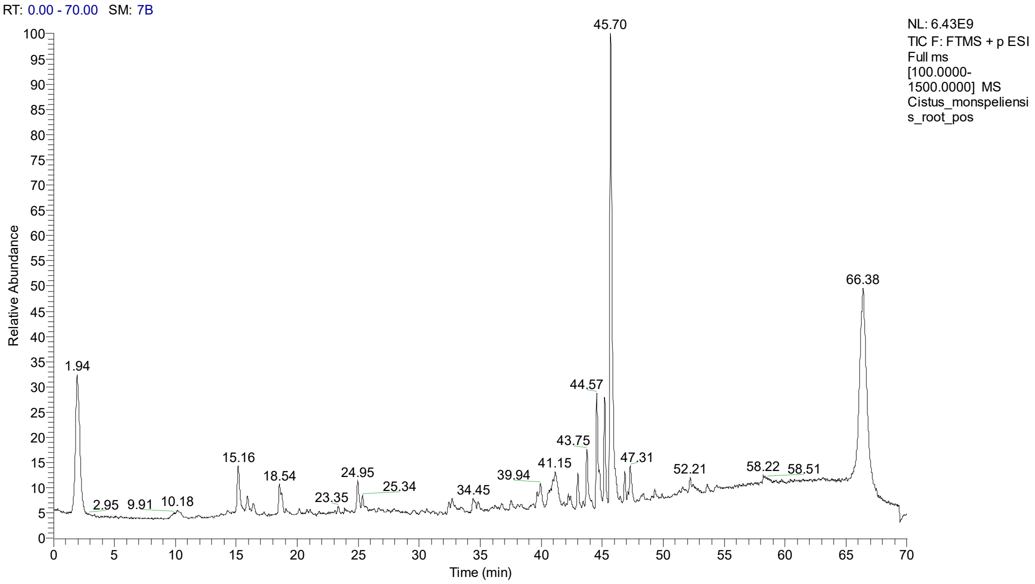

Figure S6. Cistus monspeliensis root TIC negative

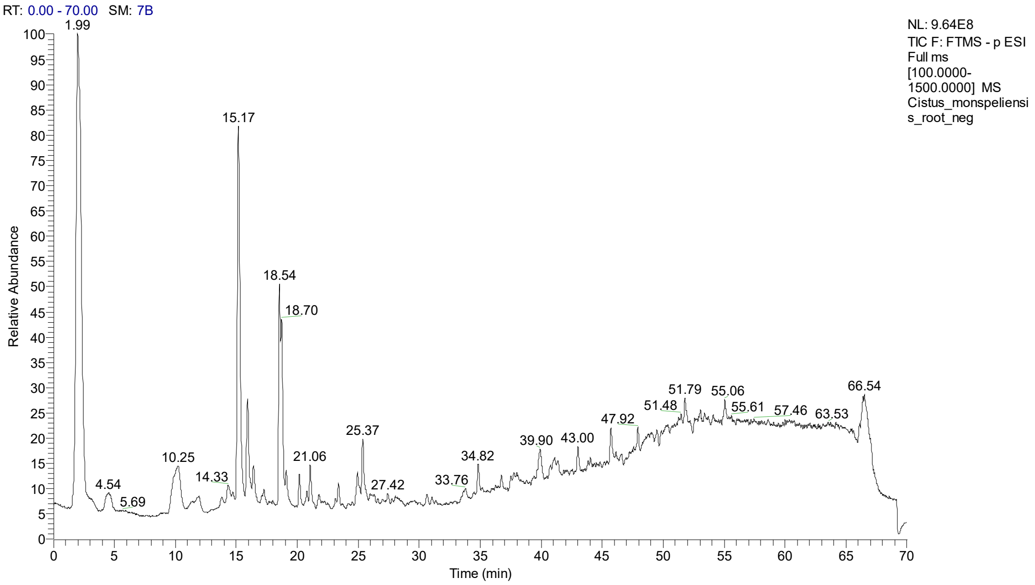

Figure S7. Cistus parviflorus leaves TIC positive

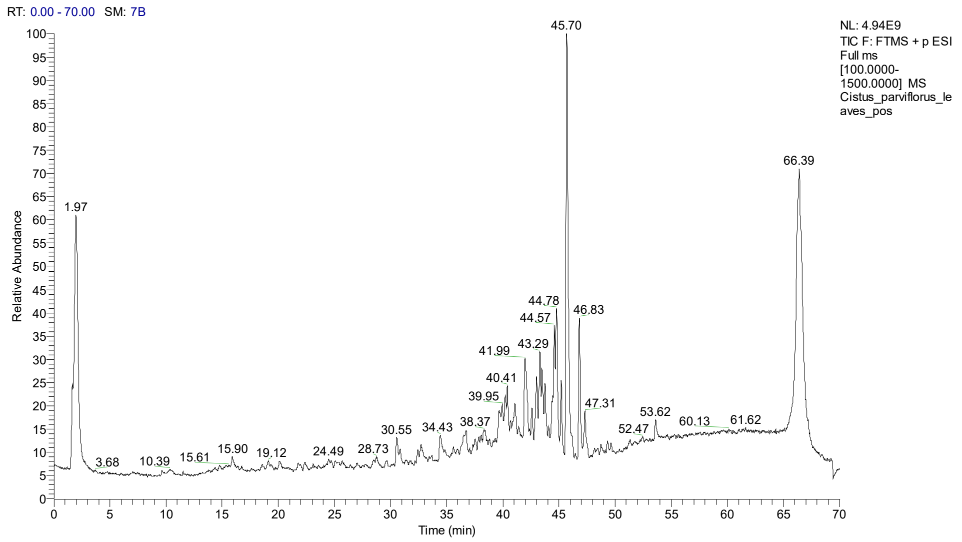

Figure S8. Cistus parviflorus leaves TIC negative

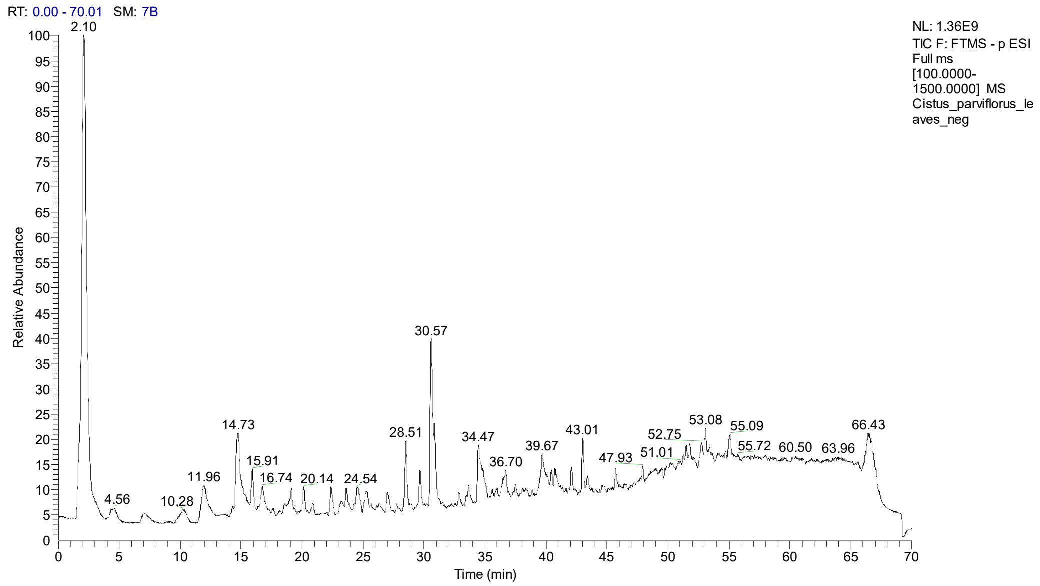

Figure S9. Cistus parviflorus twigs TIC positive

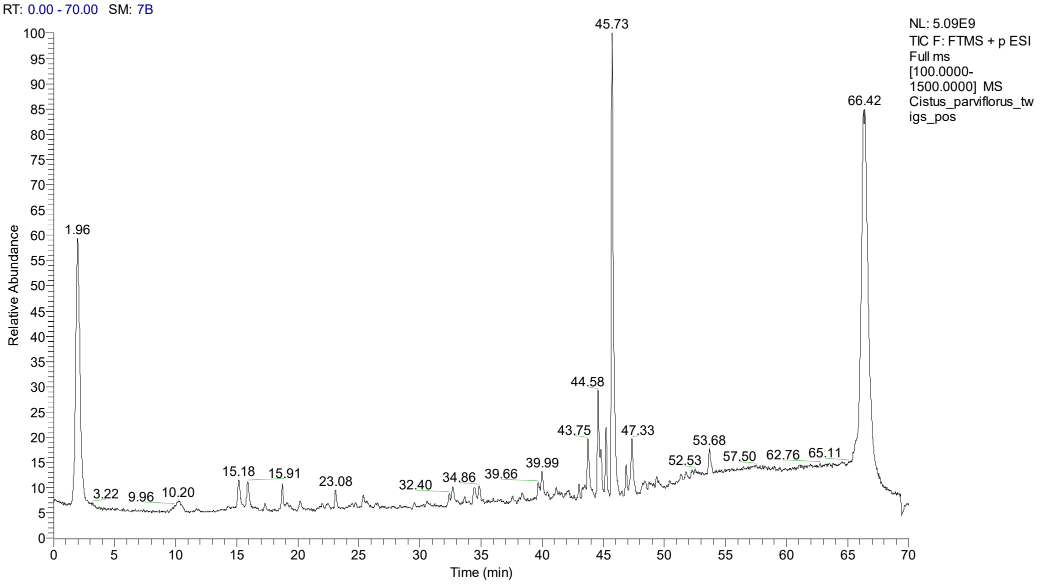

Figure S10. Cistus parviflorus twigs TIC negative

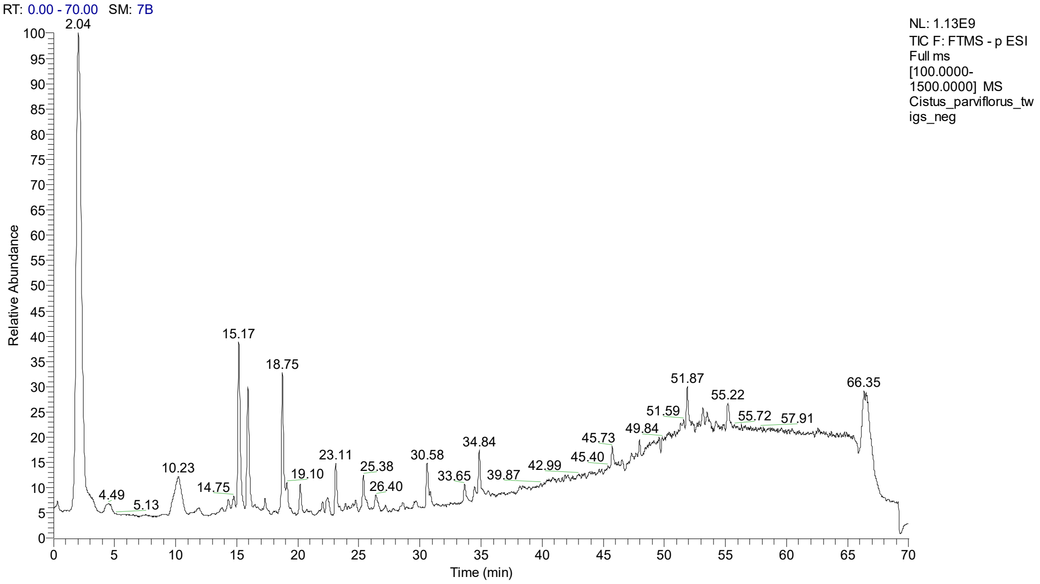

Figure S11. Cistus parviflorus root TIC positive

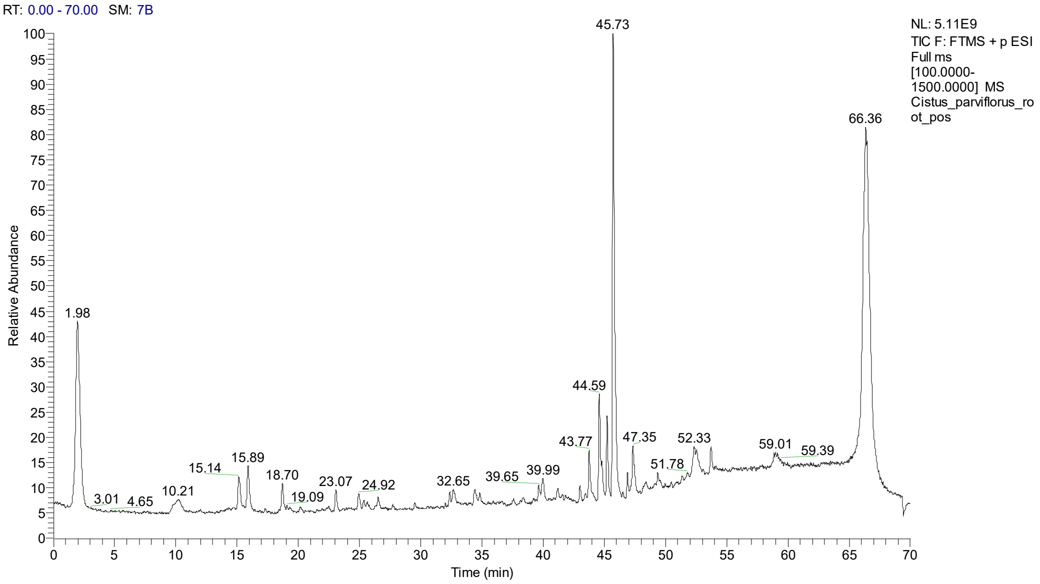

Figure S12. Cistus parviflorus root TIC negative

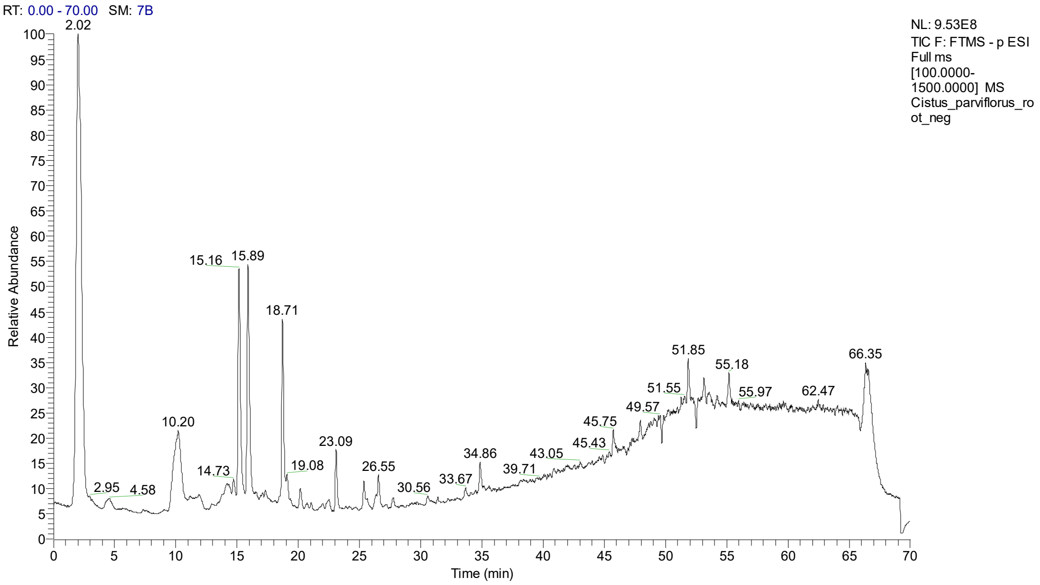

Supplement: Supplementary file 1 [file pathogens-13-00795-s001.zip › pathogens-3192420-supplementary.pdf]
